# Targeting bromodomain and extra-terminal proteins reprograms macrophages and inhibits breast cancer progression

**DOI:** 10.3389/fimmu.2026.1738705

**Published:** 2026-09-03

**Authors:** Deeksha Sharma, Cody G. Hager, Grace G. Bushnell, Alexander P. Kalman, Grace F. Shapiro, Katherine A. Xendzova, Lynne Walenjus, Chloe M. Hutchens, Max S. Wicha, Monika L. Burness

**Affiliations:** 1 University of Michigan Rogel Cancer Center, Internal Medicine, Ann Arbor, Michigan, IN, United States; 2 University of Minnesota, Biomedical Engineering, Minneapolis, MN, United States

**Keywords:** BETd (Bromodomain and extra-terminal protein degrader), breast cancer, cancer stem cell, single-cell RNA sequencing, TAM (tumor-associated macrophage)

## Abstract

**Introduction:**

Breast cancer (BC) remains a leading cause of cancer-related deaths, with resistance to therapy driving tumor progression. While immune checkpoint inhibitor (ICI) therapy is effective in some breast cancers, the heterogeneity of treatment responses is partly driven by cancer stem cells (CSCs) and an immunosuppressive tumor microenvironment (TME). Bromodomain and extra-terminal (BET) proteins regulate tumor and immune biology, but their role in innate anti-tumor immunity remains poorly understood. We investigated the role of BET proteins in modulating innate immunity using the potent BET degrader (BETd) ZBC260.

**Methods:**

ZBC260 efficacy was evaluated in immunocompetent BALB/c mice bearing D2A1 tumors. NK cells or macrophages were depleted to determine their contribution to BETd response. *In vitro*, primary murine NK cells, bone marrow-derived macrophages, and THP-1, NK-92 MI, SUM-149, and D2A1 cells were used for co-culture, transwell migration, phagocytosis, qPCR, ELISA, and flow cytometry assays. Single-cell RNA sequencing (scRNA-seq) was performed using 10x Genomics 3′ sequencing and analyzed with Seurat.

**Results and discussion:**

ZBC260 inhibited tumor growth in SCID mice but not in NOD-SCID mice, with impaired macrophages and partially compromised NK cells, implicating the innate immune system. To assess the contribution of innate immune cells to BETd efficacy, we depleted NK cells or macrophages in immunocompetent BALB/c mice with D2A1 tumors and treated with ZBC260 or vehicle. Immune depletion studies revealed macrophage loss partially attenuated anti-tumor and anti-CSC effects, highlighting their critical role. To understand the impact of BETd on TME populations, we performed single-cell RNA sequencing, which identified myeloid cells as the predominant population. BETd reduced MRC1⁺immunoregulatory tumor-associated macrophages and enriched CD74+ antigen-presenting macrophages, with upregulated phagosome pathways and enhanced Stat1 activation. *In vitro*, BETd downregulated M2-associated markers, promoted macrophage phagocytosis, induced tumor cell apoptosis, and inhibited macrophage-driven CSC formation. Collectively, these findings demonstrate that BETd ZBC260 reshapes the TME and reprograms macrophages toward an anti-tumor phenotype, highlighting its translational potential for breast cancer therapy.

**Conclusion:**

Collectively, these findings demonstrate that BETd ZBC260 reshapes the TME and reprograms macrophages toward an anti-tumor phenotype, highlighting its translational potential for breast cancer therapy.

## Introduction

Breast cancer (BC) remains the most prevalent malignancy and the leading cause of cancer-related morbidity, disability, and mortality among women globally (1). Despite significant advancements in therapeutic strategies, overcoming resistance to conventional treatments remains a critical challenge (2). Newer approaches, including immunotherapies, have shown remarkable promise in improving patient outcomes. However, the complex biology of BC and the presence of cancer stem cells (CSCs), a population characterized by self-renewal ability and therapy resistance, limit the efficacy of these novel approaches particularly in aggressive subtypes like triple-negative breast cancer (TNBC) (3–5). Breast CSCs are commonly identified by phenotypic markers such as CD44^+^/CD24^-^ expression and elevated aldehyde dehydrogenase (ALDH) activity, both of which are associated with tumor-initiating capacity and therapy resistance (6).

Epigenetic regulators play a significant role in oncogenesis, with BET (bromodomain and extra-terminal domain) proteins acting as key regulators of CSC self-renewal and tumorigenicity (7, 8). Inhibition of BET proteins suppresses super-enhancer-associated oncogenes and disrupts inflammatory signaling, thereby impairing tumor growth and stemness (9–12). BET proteins also play a vital role in regulating the immune response to tumors by modulating the tumor microenvironment (TME). They affect multiple cell types, including boosting the cytotoxic activity of natural killer (NK) cells against tumor cells and decreasing the proliferation of tumor-associated macrophages (TAMs) (13, 14). Additionally, BET inhibition improves the effectiveness of ICI therapy and chemotherapy in preclinical models of BC (15). Previous studies have shown that the pan-BET degrader (BETd) ZBC260 exhibits potent and selective pan-BET degradation activity (16, 17). Building on these findings, our recent work demonstrated that ZBC260 exerts robust anti-tumor and anti-stemness effects in breast cancer models [9], supporting its use in the present study to investigate the impact of BET protein degradation on the tumor microenvironment (TME). In the complex landscape of the TME, innate immune cells including NK cells and macrophages are regulators of BC progression (18). NK cells are critical mediators of immune surveillance, and their dysfunction contributes to tumor progression, metastasis, and dormancy (19, 20). In addition to NK cells, macrophages represent one of the most abundant and functionally diverse immune populations within the TME. Their high plasticity allows them to adopt distinct functional states shaped by their ontogeny, tissue context, and microenvironmental signals (21). Although they have historically been classified into M1-like (pro-inflammatory, anti-tumor) and M2-like (immunoregulatory, pro-tumor) states, this binary framework does not fully capture the diversity of TAM phenotypes observed in tumors (22). Recent advances in single-cell RNA sequencing (scRNA-seq) and spatial transcriptomics have demonstrated that TAMs exist along a continuum of functionally distinct states, with transcriptional programs related to immunoregulation, antigen presentation, interferon responses, and tissue remodeling across multiple cancer types, including breast cancer (23–25). Mechanistically, TAMs support tumor growth by secreting pro-tumor cytokines, suppressing T cell activity, and promoting tumor resistance via the JAK/STAT and MAPK pathways (26–28). Current therapeutic strategies focus on limiting macrophage recruitment, disrupting tumor-supportive signaling, and reprogramming TAM functional states to enhance antigen presentation and immune activation; among these, modulation of TAM functional states has emerged as a particularly promising approach (29).

In the present study, we utilized ZBC260 to explore the effects of BETd on the innate immune system and determine the mechanisms behind these effects. Using *in vivo* mouse models as well as *vitro* assays, we showed that BETd reprograms macrophage functional states by reducing tumor-supportive phenotypes and enhancing antigen-presenting and tumoricidal functions.

Additionally, BET degradation enhanced macrophage-mediated tumor cell apoptosis and subsequent phagocytosis. The use of this compound, which targets and degrades BET proteins across all cell types within the TME, offers significant advantages for our investigations versus cell type-specific genetic manipulation. This approach enables the assessment of complex and dynamic intercellular interactions within the TME, thereby enhancing the translational relevance of the findings. Overall, our findings underscore the importance of macrophage-driven anti-tumor immunity and suggest that BET-targeted therapies could enhance immunotherapy strategies and outcomes for BC.

## Materials and methods

### Cell culture and maintenance

SUM149 cell line (RRID#CVCL_3422) provided by Dr. Stephen Ethier (Karmanos Cancer Institute, USA). D2A1 cell line (RRID#CVCL_0I90) provided by the laboratory of Hunter Kent (National Cancer Institute, USA (20), and the THP-1 (RRID#CVCL_0006) and NK-92MI (RRID#CVCL_3755) cell lines were provided by the laboratory of Dr. Leo Lei (University of Michigan, USA) (20, 30). SUM149 cells were maintained in F-12 media (Cat#1765064, Invitrogen) supplemented with 5% fetal bovine serum (FBS), 5 µg/mL insulin (Cat#I2643, Sigma), 1 µg/mL hydrocortisone, 1x penicillin/streptomycin (pen/strep) (Cat#15140122, Gibco) [9]. D2A1 cells were maintained in DMEM (Cat#11960044, Gibco) with 10% FBS and 1x GlutaMAX (Cat#35050061, Gibco) and 1x Pen/strep (20). NK-92MI cells were maintained in MEM media (Gibco), with 10% FBS, 1x pen/strep (Gibco), 1x GlutaMAX (Gibco), 0.1mM 2-mercaptoethanol, 0.2 mM inositol, 0.02 mM folic acid. THP1 cells were maintained in RPMI 1640 (Gibco) with 10% FBS and 1x Pen/strep. Cells were monitored regularly for mycoplasma contamination using the MycoAlert Mycoplasma Detection Kit (Cat#LT07-318, Lonza) (authenticated by ATCC).

THP-1 cells were differentiated into THP-1 macrophages (THP-1 M0) using 150 ng/ml phorbol 12-myristate 13-acetate for 72 hours (PMA) (Cat#P8139, Sigma). These differentiated cells were polarized into M1 macrophages after 24 hours of PMA treatment with 20 ng/ml of Interferon-γ (IFN-γ) (Cat# 30002, Peprotech) and 10 pg/ml of lipopolysaccharide (LPS) CAT# 8630, Sigma) for next 48 hours. Similarly, M2 macrophage polarization was obtained by incubation with 20 ng/ml of interleukin 4 (IL4) (Cat# 20004, Peprotech) and 20 ng/ml of interleukin 13 (IL13) (Cat# #20013, Peprotech) (30). Primary murine NK cells (mNK) were isolated from the spleens of naïve BALB/c mice (Cat#000651, RRID#IMSR_JAX:000651, Jackson Laboratory) (6–10 weeks old) and cultured with RPMI1649 media (Gibco) with 10% FBS, murine recombinant, 1x Pen/strep, Interleukin-2 (IL-2) (Cat#212-12, Peprotech) and β-mercaptoethanol, as previously described (20).

Primary murine macrophages were isolated from the bone marrow of BALB/c mice. Bone marrow was harvested from the femurs and tibias of 6–10-week-old BALB/c mice. Briefly, femurs and tibias were excised at the hip joint, cleaned of surrounding skin and muscle tissue, and the metaphyses were removed at the knee to expose the marrow cavity. Bones were placed marrow side down in a 0.5 milliliter microcentrifuge tube with a small hole in the bottom. This tube was placed in a larger 1.5 milliliter tube and centrifuged at 10,000 × g for 15 seconds to remove the bone marrow cells from the bones. Successful centrifugation was confirmed by a large red pellet at the bottom of the larger tube and a white appearance of the bones in the top tube. Bones were discarded and marrow cells were resuspended in sterile PBS, then processed and cultured in DMEM media (Gibco 11960044) supplemented with 10% FBS, 1x pen/strep, and 50 ng/mL murine colony stimulating factor (mCSF) (Cat#250-05, Peprotech) for 5 days to generate bone marrow macrophages (BM-macrophages) (20, 31, 32). On day 6 post-isolation, BM-macrophages were polarized to M1 macrophages (BM-M1) using IFN-γ (20 ng/mL) and LPS (10 pg/mL) or to M2 macrophages (BM-M2) using IL-4 (25 ng/mL) and IL-13 (25 ng/mL) for 24 hours, following established protocols (31, 32).

### Chemical reagents

ZBC260 was provided by Dr. Shaomeng Wang Lab (Department of Pharmacology, University of Michigan) and purchased (Cat#HY-101519, MedChem Express) (9).

### Cell viability and clonogenic assay

Cell viability of adherent cells (D2A1, THP-1-derived macrophages, and bone marrow-derived macrophages) was assessed using the MTT assay (3-(4,5-dimethylthiazol-2-yl)-2,5-diphenyl-2H-tetrazolium bromide; Cat#00697, Chem-Impex Intl) (9). Briefly, 10,000 D2A1 cells were plated overnight in a 96-well low-evaporation plate and treated with ZBC260 at concentrations of 1.56, 3.125, 6.25, 12.5, 25, and 50 nM for 72 hours. THP-1- and bone marrow-derived macrophages were first differentiated and polarized, then plated in similar conditions and treated with ZBC260 for 72 hours. Following treatment, cells were incubated with MTT reagent (Cat#M2128, Sigma-Aldrich) at 37 °C for 3 h, and absorbance was measured at 560 nm using a microplate reader. NK92MI cells were plated as a suspension and treated with the same ZBC260 concentrations for 24, 48, 72, 96, and 120 hours. Cell viability was assessed using the CellTiter-Glo assay (Cat#G9681, Promega) according to the manufacturer’s instructions.

The effect of NK92-MI and mNK cells on clonogenic potential (colony formation) of SUM149 and D2A1 respectively was measured using crystal violet staining. Briefly, 1000 cells/well for D2A1 and 2000 cells/well for SUM149 were plated in 6 well plates and allowed to attach overnight then mNK (5000/well) or NK92MI in (10000/well) were added with vehicle or ZBC260 (25nM) for 72hours. After 72 hours media was removed and NK cells were washed twice with PBS, followed by addition of new media. Then cells were allowed to grow for an additional 8 days. Cells were then fixed with 4% paraformaldehyde and stained with 0.5% crystal violet. The assay was performed three times for each treatment. Colonies with more than 50 cells were counted under microscope.

### NK cell cytotoxicity assays

NK cytotoxicity was assessed by co-culturing Calcein-AM-labeled (Cat#65-0853-78, Invitrogen) SUM149 and D2A1 cells with NK-92MI or mNK cells (1:5 ratio) for 24 hours, with vehicle or 25nM ZBC260. Cytotoxicity was measured using a fluorescence plate reader (Cat#H1MG, RRID#SCR_019748, BioTek), quantifying viable cells by relative fluorescence intensity (RFU, 490 nm excitation, 520 nm emission), and calculating the percentage cytotoxicity:

(1) NK Cell Cytotoxicity (%) = [(Fluorescence of target cells alone − Fluorescence of co-cultured cells)/Fluorescence of target cells alone] × 100.

### Fluorescent lipid labeling

SUM149, D2A1, and macrophages were stained with Lipophilic membrane dyes DiO (Cat#D275, Invitrogen), DiI (Cat#D3911, Invitrogen), and DiD (Cat#V22887, Invitrogen) (2.5 µg/ml) for transwell migration, co-culture, and tumor infiltration assays (manufacturer’s instructions).

### Phagocytosis assay

DiI-labeled differentiated THP-1 or BM-macrophages were co-cultured with DiO-labeled SUM149 or D2A1 cells (1:2 ratio) and treated with vehicle or 25 nM ZBC260. Phagocytosis was monitored over 72 hours using the IncuCyte ZOOM (RRID#SCR_026298), with images captured every 2 hours and fluorescence overlap used to quantify phagocytosis. Flow cytometry (FITC for tumor, PE for macrophages) identified dual-positive (phagocytic) cells, using monocultures and unlabeled cells as controls. As a complementary assessment of macrophage-mediated tumor-cell uptake co-cultures were imaged using an EVOS FL fluorescence microscope (Thermo Fisher Scientific; Cat#AMF-4300, RRID). Images were acquired using identical exposure settings across all experimental conditions. Three independent biological experiments were performed, with minimum three technical replicates per condition in each experiment. Five randomly selected fields of view were analyzed for each technical replicate condition. Representative images and quantitative analyses were performed using images acquired with a 10× objective. Fluorescence images were analyzed using QuPath software automated script (Data Availability Section). DiI-positive macrophages were identified, and DiO fluorescence within macrophage boundaries was quantified as a measure of tumor cell uptake. Macrophages containing DiO-positive fluorescence above background levels were classified as DiO-positive/phagocytic macrophages. The percentage of DiO-positive macrophages and intracellular DiO fluorescence intensity per macrophage were calculated and compared between treatment groups.

Macrophage phagocytic activity was assessed using the IgG-FITC Phagocytosis Assay Kit (Cat#500290, Cayman Chemical). Macrophages were incubated with FITC-labeled rabbit IgG-opsonized latex beads supplied with the kit in the presence of ZBC260 (25 nM) or vehicle control for 24 hours, according to the manufacturer’s instructions. Bead uptake was evaluated by fluorescence microscopy (EVOS FL, 4× objective; Cat#AMF-4300) and quantified by flow cytometry using a Bio-Rad ZE5 Cell Analyzer (Cat#12004278). Unstained macrophages and FITC-bead-only controls were included to establish gating parameters and assess background fluorescence. FCS files were analyzed with FlowJo software (v10.8.0, RRID#SCR_008520).

### Apoptosis

Vehicle or 25 nM ZBC260 treated co-cultured cells with monoculture control were stained with Annexin-V-APC (Cat#550475, RRID#AB_2868885, Pharmingen) and PI (Cat#421301, Bio Legend), the percentages of apoptotic cells (Annexin-V^+^ DAPI+) were determined by flow cytometry (ZE5, Bio-Rad).

### Tumorsphere infiltration and CSC analysis

Tumorsphere formation assays were performed using MammoCult Basal Medium (Cat#05620, STEMCELL Technologies) (9). DiO-labeled SUM149 cells were co-cultured with DiI-labeled NK-92MI or THP-1-macrophages (M0, M1, M2) for 14 days with vehicle or 25 nM ZBC260. At the experimental endpoint, tumorspheres were counted and macrophage infiltration imaged using 4x magnification on the EVOS FL microscope. ALDH^+^ CSC markers were evaluated using the ALDEFLUOR assay (ALDEFLUOR™ Kit, Cat#01700, STEMCELL Technologies) with DiI-stained SUM149 cells and DiD-stained macrophages. For CD24^-^/CD44^+^ CSC analysis, SUM149 cells were stained with DiO. Co-cultures were maintained at a 1:2 ratio (SUM149: THP-1-M0) for 14 days. After dissociation, cells were stained for CD24 and CD44 markers and analyzed by flow cytometry. Antibodies used, with catalog# and RRID#, are listed in **Supplementary Table 1**.

### Conditioned media

THP-1 M0, THP-1 M1, and THP-1 M2 macrophages, as well as SUM149/D2A1 cells were treated with vehicle or 25 nM ZBC260 for 48 hours, washed, and then incubated in serum-free media for 48 hours. The conditioned media (CM) were subsequently collected, filtered through a 0.22 μm filter and stored at -80 °C until further use.

### Transwell migration assay

Migration assays were performed in 6.5 mm Transwell plates with 8 μm pore inserts. THP-1 M0, THP-1 M1, THP-1 M2 macrophages, NK-92MI cells, BMMs (BM M0, BM M1, BM M2), and mNK were treated with 25 nM ZBC260 for 24 hours, labeled with DiI, and seeded in the upper chamber with serum-free media, while SUM149 or D2A1 conditioned media (CM) was placed in the lower chamber. After 48 hours, migrated cells were visualized (4x magnification, Evos FL) and quantified using QuPath software (RRID#SCR_018257) with appropriate controls.

### RNA isolation and qPCR

Gene expression analysis was done in differentiated and polarized THP-1 cells treated with vehicle or 25 nM ZBC260 for 24 hours as previously described (9). Primers are listed in **Supplementary Table 2**. Each sample was run in triplicate, and target gene expression levels were normalized to GAPDH and analyzed using the ΔΔCt method.

### Enzyme-linked immunosorbent assay

ELISA was performed by the Immune Monitoring Core of the Rogel Cancer Center (RRID#SCR_025765) using ELISA kits (Duoset, R&D Systems) on THP-1-macrophages.

### Western blotting

Vehicle and 25 nM ZBC260 treated THP-1-macrophages, D2A1, and NK-92MI cells were analyzed for protein expression via western blotting as previously described (9). Band density of target proteins was normalized to actin band density (loading control). Antibodies used, with catalog# and RRID#, are listed in **Supplementary Table 1**.

#### Multiplex immunofluorescence staining

Multiplex immunofluorescence (mIF) was performed on formalin-fixed paraffin-embedded (FFPE) blocks from CB17-SCID mice (Cat#236, RRID#IMSR_CRL:236, Charles River) using antibodies against CD45 (Opal 690, 1:5000), pan-cytokeratin (Opal 480, 1:200), F4/80 (Opal 520, 1:500), and CD49b (Opal 780, 1:1000). Staining was done on Ventana Discovery Ultra Stainer (RRID#SCR_021254, Ventana Medical Systems, Inc.) at the Tissue and Molecular Pathology Core of University of Michigan (RRID#SCR_026803). Detection used Ventana Omnimap anti-rabbit HRP and Opal Tyramide Signal Amplification, with antigen retrieval via Ultra CC2 buffer (RRID#950-223, Roche). Slides were mounted with ProLong Gold/DAPI (Cat#P36941, Invitrogen) and imaged at 20x using the Akoya Polaris IF Scanner (Cat# CLS143455, RRID#SCR_025508). Antibodies used, with catalog# and RRID#, are listed in **Supplementary Table 1**.

### Animal studies

All animal studies were approved by the University of Michigan Institutional Animal Care and Use Committee (IACUC) (PRO00011242, RRID#SCR_025789), performed in accordance with relevant guidelines and regulations, and conducted in a pathogen-free facility (9). Female six-week-old NOD-SCID (RRID#IMSR_JAX:001303) and BALB/c mice were orthotopically injected with 5 × 10⁵ SUM149 or D2A1 cells into the fourth left mammary fat pad. Once tumors reached 200 mm³, mice (n=10 or 15/group) were randomized to receive ZBC260 (5 mg/kg) or vehicle (20% PEG400, 6% Cremophor EL) intravenously, three times per week. For NK cell depletion, BALB/c mice received 300 µg anti-Asialo GM1 (anti-ASGM1) antibody (Cat#146002, RRID#AB_2562206, BioLegend) or normal rabbit serum (Cat#ab7487, RRID#AB_2753117, Abcam) intraperitoneally every four days. The anti-ASGM1 dose used in this study was selected based on previously published protocols using the same product as well as our prior optimization (Bushnell et al.,2024). For macrophage depletion, BALB/c mice received 2 mg clodronate-containing liposomes or empty liposomes (Cat#CLD-8901, Encapsula NanoSciences) intraperitoneally every four days. Mice were euthanized when tumors exceeded 2 cm. Euthanasia was performed by CO₂ displacement at a flow rate of 40% of chamber volume per minute (0.0432 L/min), and death was confirmed by an approved secondary physical method (induction of bilateral pneumothorax or decapitation) in accordance with the University of Michigan’s IACUC approved protocol.

All mice/data points were included in the study. Tumor size and body weight were monitored twice weekly for all mice/experiments. Sample sizes were based on prior experience with the experimental models and are consistent with commonly accepted standards in the field for detecting biologically meaningful differences. Randomization was performed using a randomizer to assign mice to experimental groups. To minimize potential confounders experiments were performed at consistent times of day and treatment was administered in a rotated sequence. No blinding was performed; all authors were aware of group assignments during experiments and analysis. No formal tests of statistical assumptions were conducted. Statistical methods were selected based on the type and distribution of the data.

Tumors were dissociated using 1X collagenase/hyaluronidase (Cat#07912, STEMCELL Technologies). Spleen and bone marrow were collected to confirm NK and macrophage depletion by flow cytometry. NK cells (CD45^+^/CD49b^+^) and macrophages (CD45^+^/F4/80^+^) were identified using fluorophore-conjugated antibodies with isotype controls and stained for 60 minutes at room temperature in the dark. Antibodies used, with catalog# and RRID#, are listed in **Supplementary Table 1**.

### Limiting dilution reimplantation assay

Dissociated bulk tumor cells from each treatment group in the macrophage depletion experiment were pooled for isolation and sorted using a mouse tumor cell isolation kit (Cat#130-110-187, Miltenyi Biotec), and reimplanted at 50, 500, or 5000 cells in the 4^th^ left and right mammary fat pad. Tumor formation was monitored for 60 days, and Extreme Limiting Dilution Analysis (ELDA) (RRID#SCR_018933) was used to calculate the average tumor initiating cell (TIC) frequency.

### Single cell RNA sequencing

FFPE tumor sections were processed for single-cell sequencing (scRNA-seq) using the 10x Genomics platform with the Single Cell 3′ Reagent Kits v3 (Cat# PN-1000121). Libraries were prepped using the NovaSeq 6000 S4 Reagent Kit v1.5 (Cat#20028312), for sequencing on Illumina NovaSeq 6000 platform (RRID#SCR_016387). Raw gene expression matrices were generated with Cellranger (v8.0.1, RRID#SCR_023221) and analyzed in Seurat (v5.2.0, RRID#SCR_016341) using Seurat vignette (https://satijalab.org/seurat/articles/pbmc3k_tutorial) (33). Cell types were annotated with the scCATCH package and known markers. Myeloid cells were further reclustered. Differentially expressed genes (DEGs) were identified using FindAllMarkers (Wilcoxon test, ≥25% expression, log2FC ≥0.5, adjusted p < 0.05). Pathway enrichment (KEGG) conducted with enri chr (v3.2, RRID#SCR_001575). SCENIC was used for transcription factor analysis, and CytoTRACE (RRID#SCR_022828) was used to calculate CSC scores. Phagocytosis, M1, and M2 scores were determined using AddModuleScore (34). Cell-cell communication was inferred using *CellChat* (RRIC#SCR_021946).

### Statistical analysis

Statistical analysis was performed using GraphPad Prism software 10 (RRID#SCR_002798, San Diego, CA) and presented as mean ± standard error of the mean (SEM). Statistical significance was determined using Student’s t-tests or one-way/two-way ANOVA or Wilcoxon test in R. P values were considered significant if *P* < 0.05.

### Graphical figures

Schematic figures created using BioRender.com (RRID#SCR_018361).

## Results

### Macrophages, not NK cells, mediate the anti-tumor and anti-CSC activity of BETd ZBC260 in breast cancer

**Figure 1:**
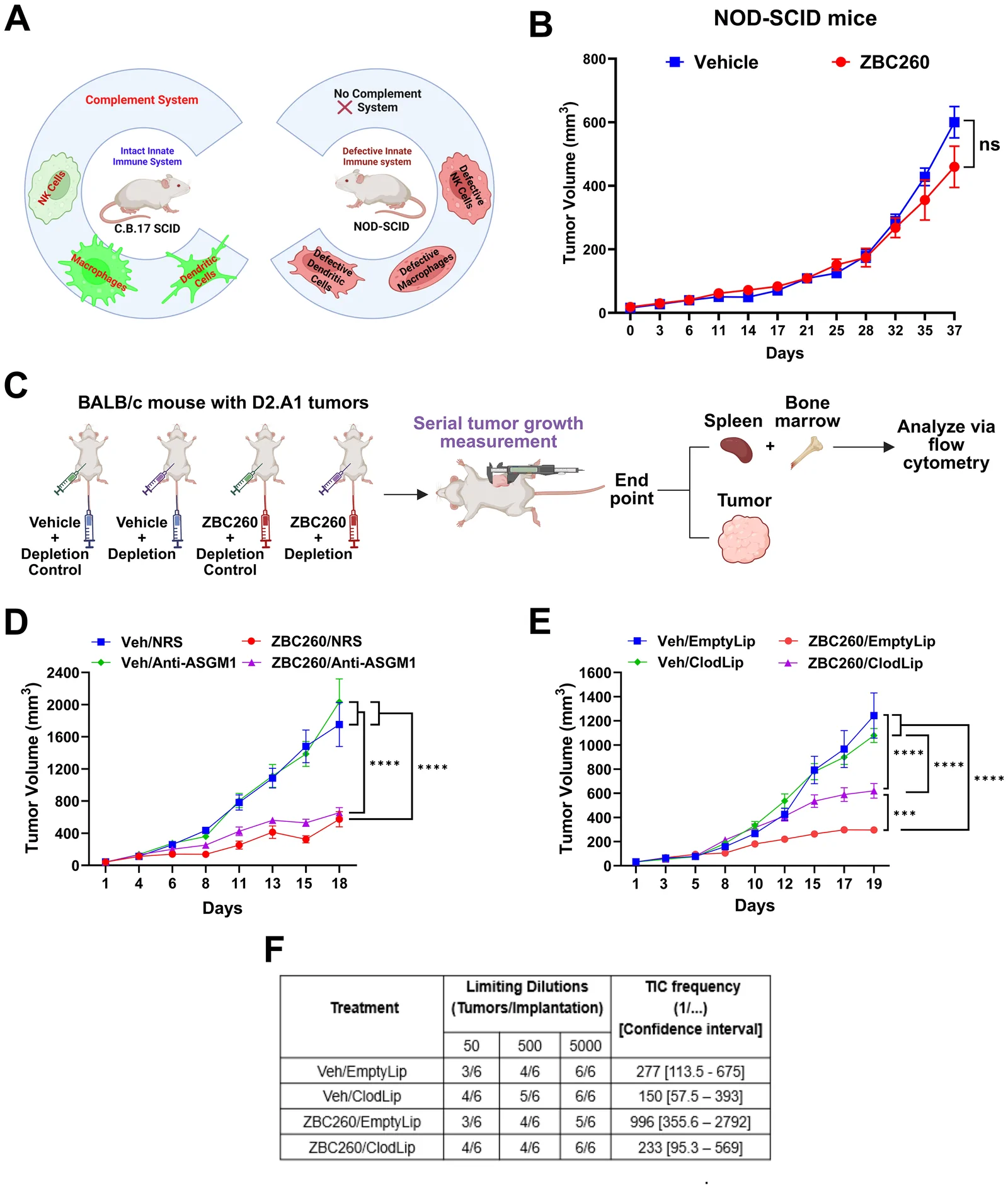
Macrophages, but not NK cells, mediate the anti-tumor and anti-CSC activity of BETd ZBC260 in breast cancer. **(A)** Schematic showing intact and defective innate immune system mouse models. **(B)** Effect of ZBC260 (5mg/kg) or vehicle on SUM149 tumor volume in NOD-SCID mice, n=15. **(C)** Schematic showing the tumor growth assay *in vivo* to examine the role of immune cells in ZBC260 efficacy. **(D, E)** Effect of ZBC260 (10 mg/kg) or vehicle on D2A1 tumor volume in BALB/c mice in combination with NRS/Anti-ASGM1 **(D)** or EmptyLip/ClodLip **(E)** as an average, n=10. **(F)** Table showing the tumor initiation frequency, calculated by ELDA, of reimplanted tumors after ZBC260 or vehicle treatment in control or macrophage-depleted mice, n=5. Extreme limiting dilution analysis was performed to calculate TIC frequency via ELDA analysis. In **(B, D, E)**, data points are the mean ±SEM for individual experiments. Data was analysed using Student’s t-tests with Welch’s correction. Data is presented as biological replicates and was analysed by two-way ANOVA with Bonferroni correction. ***p<0.001, ****p<0.0001.

We previously reported that BETd ZBC260 effectively suppressed breast tumor growth in CB17-SCID mice, which lack T and B cells but possess innate immune cells (9). To further dissect the contribution of immune cells, we next evaluated ZBC260 in NOD-SCID mice, which lack T, B, and macrophage cells and exhibit impaired NK cells activity (35) (**Figure 1A**). As anticipated, tumors in NOD-SCID mice exhibited accelerated growth and reached endpoint criteria sooner than in CB17-SCID mice. Unlike the efficacy observed in CB17-SCID mice, ZBC260 had no significant impact on tumor growth in NOD-SCID animals, suggesting that intact innate immune cells are required for ZBC260 efficacy (**Figure 1B**).

To identify the relevant immune cells, we utilized the aggressive and metastatic D2A1 mammary adenocarcinoma model in immune-competent BALB/c mice. This model closely mirrors tumor progression and immune interactions within the TME (36). *In vitro*, ZBC260 degraded BET proteins in D2A1 cells and suppressed both viability and tumorsphere formation in a dose-dependent manner (**Supplementary Figures 1A–D**). We first depleted NK cells in tumor-bearing BALB/c mice (**Figure 1C**). NK cell loss, confirmed by flow cytometry (**Supplementary Figures 2A–C**), did not alter tumor growth with or without ZBC260 treatment (**Figure 1D**).

Consistently, although ZBC260 reduced BET protein levels in NK-92MI cells, it did not alter NK cell viability, cytotoxicity, colony formation, migration, or NK cell-mediated activity in tumorsphere co-culture assays (**Supplementary Figures 2D–J**). Together, these data exclude NK cells as mediators of ZBC260 therapeutic efficacy.

We next depleted macrophages using clodronate liposomes (**Figure 1C**). Macrophage loss was confirmed in bone marrow and spleen (**Supplementary Figures 2K–M**). While ZBC260 significantly suppressed tumor growth in control mice, this effect was markedly attenuated following macrophage depletion (**Figure 1E**). To assess whether ZBC260 reduces tumor-initiating cell (TIC) frequency *in vivo*, we performed Extreme Limiting Dilution Analysis (ELDA) in mice with or without macrophage depletion. In the vehicle group, TIC frequency was 1 in 277 cells and modestly decreased to 1 in 150 upon macrophage depletion. ZBC260 treatment substantially reduced TIC frequency to 1 in 996 in the presence of macrophages, however this effect was partially reversed when macrophages were depleted (1 in 233) (**Figure 1F**). Pairwise comparisons of TIC frequency between groups (Pr(>Chisq)) confirmed the significance of these differences (**Supplementary Figures 2N, O**). Collectively, these data indicate that ZBC260-mediated reduction in CSC/TIC frequency is largely dependent on macrophages. These results identify macrophages, but not NK cells, as essential mediators of the anti-tumor and anti-CSC effects of ZBC260.

### BETd ZBC260 remodels myeloid cells to enhance anti-tumor activity

**Figure 2:**
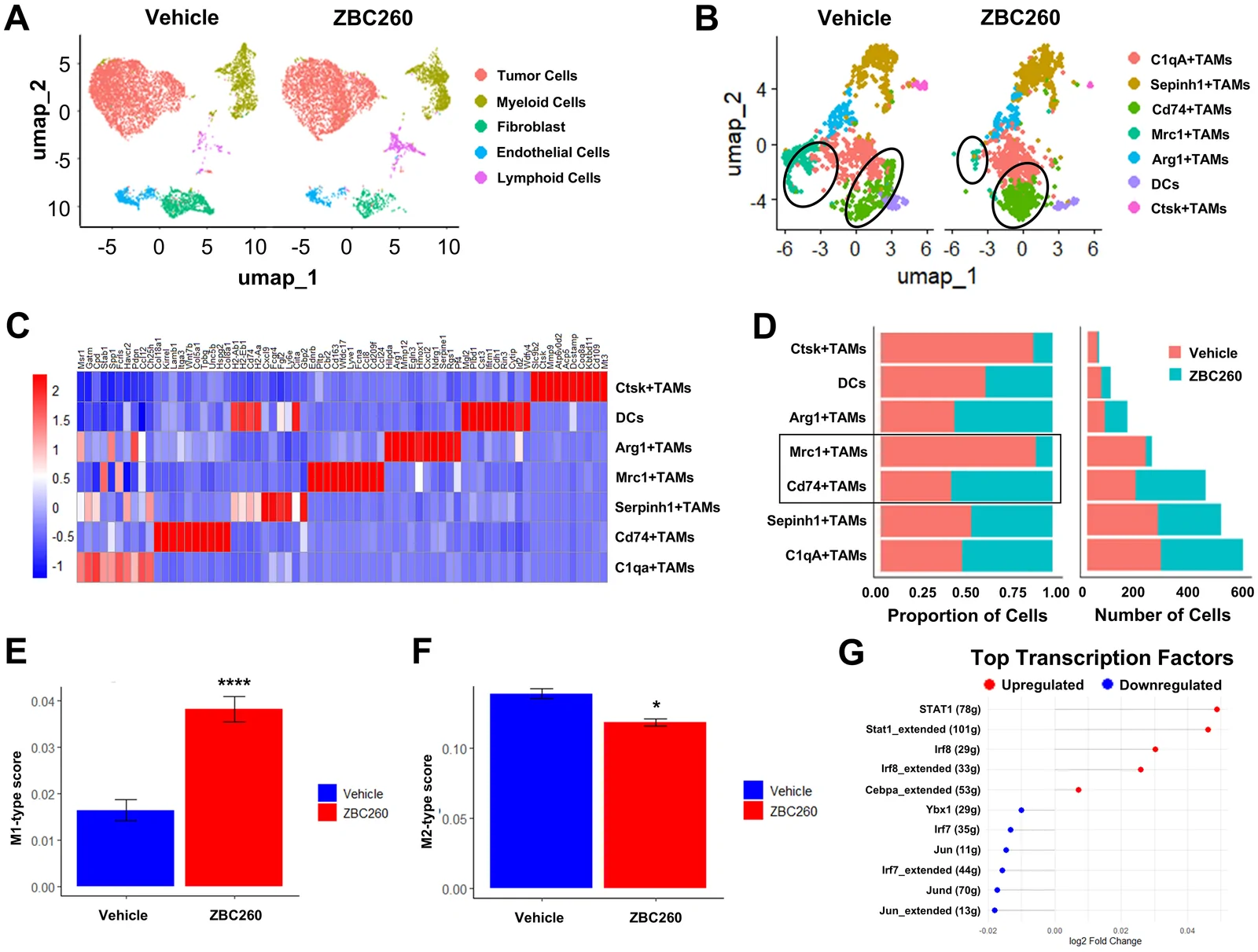
BETd ZBC260 remodels the myeloid cells to enhance anti-tumor activity. scRNA-seq analysis of *in vivo* tumor tissue from D2A1 tumor-bearing mice treated with vehicle or ZBC260 (n=3). **(A)** UMAP plot of major cell types identified in D2A1 tumors by single-cell RNA sequencing (scRNA-Seq), analysed using unsupervised clustering. Cell types were identified using known cell type-specific markers. **(B)** UMAP plot showing subtypes of myeloid cells. **(C)** Heatmap showing the top 10 markers (average expression) across the indicated myeloid subtypes. **(D)** Effect of ZBC260 treatment on the proportions and number of myeloid cell subpopulations. **(E, F)** Bar graph of M1-type and M2-type score of TAMs treated with vehicle or ZBC260. **(G)** Lollipop plot of transcription factors. Data is presented as biological replicates and technical replicates and was analysed by two-way ANOVA with Bonferroni correction. *p<0.05; ****p<0.0001.

To further define how ZBC260 modulates the tumor microenvironment, we performed scRNA-seq on D2A1 tumors from vehicle and ZBC260-treated BALB/c mice. Unsupervised UMAP-based clustering revealed that myeloid cells represented the dominant immune population in the TME under both conditions, while ZBC260 treatment increased lymphoid populations and reduced fibroblast and endothelial cells (**Figure 2A**; **Supplementary Figures 3A–D**). Given that macrophages were critical for ZBC260 efficacy *in vivo*, we focused our scRNA-seq analysis on the myeloid cells to elucidate the phenotypic and transcriptional changes responsible for this effect.

To specifically analyze macrophage populations, we performed a secondary clustering analysis of myeloid cells. This stratification identified 7 distinct subtypes, including multiple tumor-associated macrophage populations and dendritic cells (DCs). These included C1qa+TAMs (markers: Adgre1, Cd68, Msr1), Serpinh1+TAMs/proliferative TAMs (markers: Top2a, Cdkn2a, Hsp90ab1), Cd74+ antigen-presenting TAMs (markers: Cd74, Cd80, Cd86, H2-Eb1), Mrc1+ immunoregulatory TAMs (markers: Mrc1, Cd163, Lyve1), Arg1+TAMs (markers: Cd14, Arg1), DCs (DCs; markers: Id2, Mgl2, H2-Eb1), and Ctsk+TAMs (markers: Adgre1, Cd68, Acp5) (**Figures 2B, C**; **Supplementary Figures 4A–E**). Notably, neutrophils were not detected, consistent with minimal expression of canonical markers (S100a8 and S100a9) (**Supplementary Figure 4F**). Functional enrichment analysis demonstrated that these TAM subpopulations exhibit distinct and specialized biological programs. Specifically, C1qa+ TAMs were enriched for immune chemotaxis, Serpinh1+ TAMs for cell cycle regulation, and Cd74+ TAMs for antigen presentation. In contrast, Mrc1+ TAMs were associated with endocytosis, Arg1+ TAMs with metabolic and HIF-1 signaling pathways, dendritic cells with antigen processing, and Ctsk+ TAMs with oxidative phosphorylation (**Supplementary Figures 5A, B**). Together, these findings highlight the marked phenotypic and functional heterogeneity of myeloid and TAM subsets within the TME.

Although overall myeloid clustering patterns remained largely unchanged, ZBC260 induced significant compositional changes within TAM populations. Specifically, Mrc1^+^ TAMs were selectively depleted, whereas Cd74^+^ antigen-presenting TAMs were expanded, indicating a shift from immunosuppressive toward immunostimulatory states (**Figure 2D**) (37, 38). Quantitative assessment using M1-like (pro-inflammatory) and M2-like (immunoregulatory) gene expression signatures demonstrated increased M1-like scores and decreased M2-like scores in ZBC260-treated tumors, consistent with a shift toward an anti-tumor macrophage phenotype (**Figures 2E, F**). To identify potential regulators of this transcriptional reprogramming, we performed transcription factor enrichment analysis, which predicted Stat1 as a key regulator associated with this phenotypic transition (**Figure 2G**; **Supplementary Figures 5C, D**). Collectively, these data suggest that ZBC260 exerts its therapeutic effects, at least in part, by reprogramming TAMs toward an anti-tumor phenotype.

### BETd ZBC260 promotes myeloid-mediated immune activation and disrupts immunosuppressive signaling in the TME

**Figure 3:**
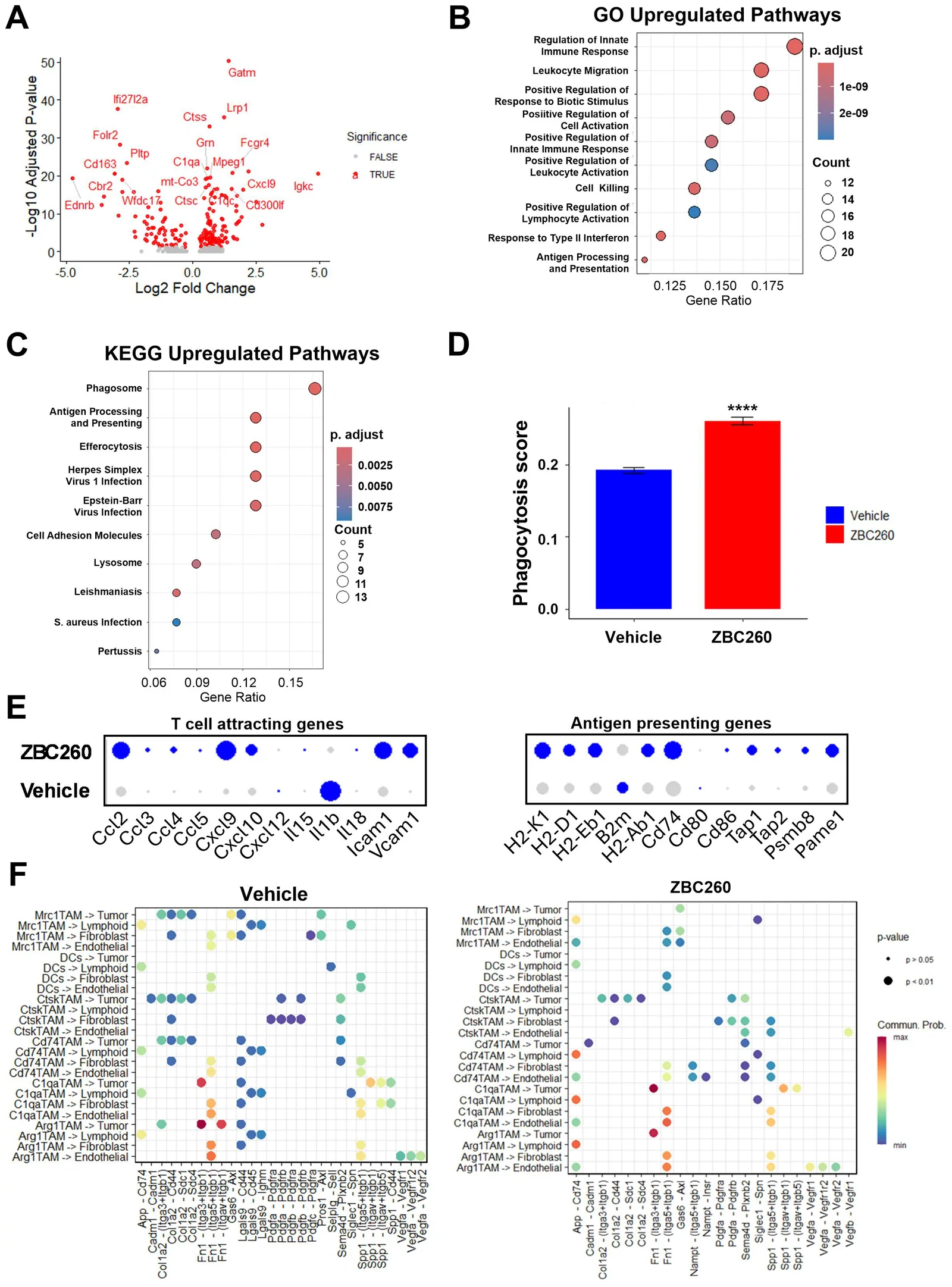
BETd ZBC260 promotes myeloid-mediated immune activation and disrupts immunosuppressive signalling in the TME. **(A–C)** Differential analysis of functional myeloid pathways in scRNA-seq data (n=3). **(A)** Volcano plot of differentially expressed genes (DEGs) between vehicle and ZBC260 treated TAMs (P < 0.05, log2(fold change) ≥ 0). **(B)** Gene ontology analysis showing the top upregulated biological pathways after ZBC260 treatment. Dot color represents log10(P) value; dot size indicates gene counts. **(C)** Dot plot of significantly upregulated KEGG pathways after ZBC260 treatment. **(D)** Bar graph of phagocytosis score of TAMs treated with vehicle or ZBC260. **(E)** Dot plot showing the effect of ZBC260 treatment on the expression of T cell-attracting genes and antigen-presenting genes. The size of the dots represents the proportion of cells expressing the particular marker. **(F)** Bubble plot showing the top ligand-receptor interaction pairs between myeloid tumor and other TME cells. Data is presented as biological replicates and technical replicates. ****p<0.0001.

To investigate how ZBC260-induced TAM reprogramming affects myeloid function, we performed differential gene expression (DGE) analysis on the myeloid cells. ZBC260 treatment significantly modulated the expression of 1,205 genes (adjusted p < 0.05), with 736 genes upregulated and 469 downregulated. Among the top 20 most significantly modulated genes, 13 were upregulated (*Gatm, Lrp1, Ctss, Grn, Fcgr4, C1qa, Mpeg1, Mt-co3, Cxcl9, Igkc, Ctsc, C1qc* and *Cd300lf*), and 7 were downregulated (*Ifi2712a, Folr2, Pltp, Cd163, Cbr2, Ednrb, and Wfdc17*) (**Figure 3A**; **Supplementary Figure 6A**).

Gene ontology (GO) enrichment analysis revealed upregulation of immune-related pathways, including ‘regulation of innate immune response’, ‘leukocyte migration’, ‘lymphocyte activation’, and ‘antigen processing and presentation’ (**Figure 3B**; **Supplementary Figure 6B**). Kyoto Encyclopedia of Genes and Genomes (KEGG) pathway analysis indicated increased phagosome formation (**Figure 3C**; **Supplementary Figure 6C**) as well as downregulation of relaxin and chemokine pathways, collectively suggesting that ZBC260 enhances both phagocytic and antigen-presenting activity in myeloid cells.

Consistent with these findings, the phagocytosis score was significantly increased in myeloid populations following ZBC260 treatment (**Figure 3D**; **Supplementary Figure 6D**). Genes involved in antigen processing and T cell activation were transcriptionally upregulated (**Figures 3E, F**), indicating ZBC260 not only polarizes TAMs toward an anti-tumor phenotype but induces gene expression associated with enhanced capacity to engage adaptive immune responses.

To assess the impact on intercellular signaling within the TME, we performed CellChat analysis, which revealed increased interactions between myeloid cells and lymphoid cells via APP-CD74, consistent with enhanced antigen presentation. In contrast, interactions mediated by FN1 and SPP1 with other TME cells were reduced, and LGALS9-mediated immunosuppressive interactions between tumor and immune cells were completely disrupted (**Figure 3F**; **Supplementary Figure 6E**). Collectively y, these findings suggest that ZBC260 functionally reprograms TAMs to enhance phagocytosis and antigen presentation, promote T cell engagement, and remodel immunosuppressive signaling within the TME, providing a mechanistic basis for the macrophage-dependent anti-tumor effects observed in **Figure 1** (38).

### BETd ZBC260 selectively promotes M1 macrophage recruitment and inhibits M2 polarization

Given the observed reprogramming of TAMs toward a pro-inflammatory phenotype, we investigated whether ZBC260 modulates macrophage infiltration, polarization, or both. To address this, we performed *in vitro* assays using human THP-1-derived macrophages and mouse bone marrow-derived macrophages (BM-macrophages).). Macrophages were pretreated with ZBC260 under M0 (unpolarized), M1, or M2 polarization conditions and then assessed for migration toward tumor-conditioned medium (**Supplementary Figure 7A**).

ZBC260 treatment did not significantly affect macrophage viability at concentrations that were cytotoxic to tumor cells (≤25 nM). Notably, this effect was polarization-dependent: all macrophage subsets exhibited reduced viability at 50 nM, whereas only M2 macrophages showed decreased viability at 25 nM. Based on these findings, 25 nM ZBC260 was selected for subsequent experiments (**Supplementary Figures 7B, C**). At this concentration, ZBC260 effectively depleted BET proteins across all macrophage subtypes (**Supplementary Figures 7D, E**). Polarization states were confirmed by increased expression of M1-type and M2-type markers (**Supplementary Figures 7F, G**).

**Figure 4:**
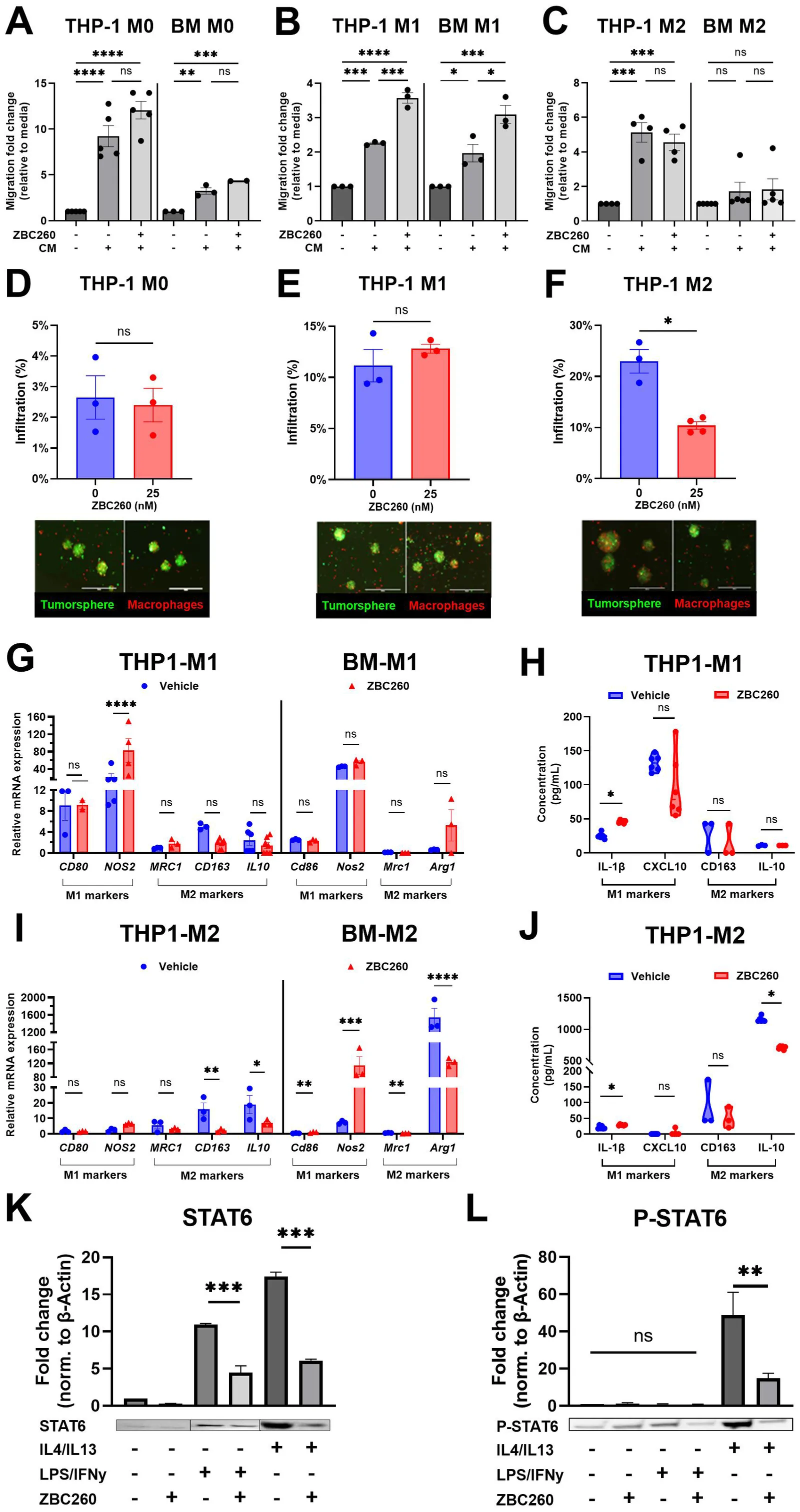
BETd ZBC260 selectively promotes M1 macrophage recruitment and inhibits M2 polarization. **(A–C)** Effect of ZBC260 pre-treatment on macrophage migration towards tumor-conditioned media compared to media alone **(A)** M0-macrophage, n=5, **(B)** M1-type macrophage, n=3, and **(C)** M2-type macrophage, n=4. **(D–F)** Quantitative analysis of macrophage infiltration of tumorspheres and fluorescence composite images showing localization of macrophages (red, DiI membrane dye) within SUM149 tumorspheres (green, DiO membrane dye) following treatment with ZBC260 or vehicle, n=3-4 (scale bar 100 μm). **(D)** Effect of ZBC260 treatment on M0-macrophage infiltration, **(E)** M1-type macrophage infiltration, and **(F)** M2-type macrophage infiltration. **(G–J)** Effect of vehicle or ZBC260 treatment on M1-type and M2-type markers in **(G, H)** M1-type macrophages and **(I, J)** M2-type macrophages, measuring **(G, I)** relative mRNA expression in THP-1 and BM macrophages and **(H, J)** cytokine expression in THP-1-macrophages. n=3–7. **(K, L)** Effect of ZBC260 or vehicle treatment on STAT6 signalling in THP-1-derived macrophages under different polarization conditions: THP-1 M0 (unstimulated), THP-1 M1 (LPS/IFNγ), and THP-1 M2 (IL4/IL13). Representatives immunoblot and quantification of **(K)** STAT6 and **(L)** phospho-STAT6 (p-STAT6), n=3–6. In **(A–J)**, data points are the mean ± SEM for individual experiments. Data is technical and biological replicates and was analyzed by Student’s t-tests with Welch’s correction **(D–J)** and one-way ANOVA with Bonferroni correction (**A–C, K, L)**. *p<0.05; **p < 0.01; ***p < 0.001, ****p < 0.0001.

Pre-treatment with ZBC260 significantly enhanced the migration of M1-polarized macrophages, whereas M2 migration was not significantly affected (**Figures 4A–C**), consistent with the selective expansion of M1-like TAMs observed previously.

Next, we established 3D tumor spheroids and quantified infiltration of differently polarized macrophages. ZBC260 treatment reduced M2 macrophage infiltration without affecting M0 or M1 infiltration, indicating that the selective M2 reduction in the TME is partly due to impaired recruitment (**Figures 4D–F**).

To assess direct effects on polarization, we measured M1 and M2 marker and cytokine expression in THP-1-macrophages and BM-macrophages. In M1-polarized cells, ZBC260 significantly increased expression of M1-type markers *NOS2*, along with increased levels of IL-1B and CXCL10 cytokines. In contrast, in M2-polarized cells, reduced the expression of M2-type markers *MRC1, CD163*, and *IL10*, and as well as decreased secretion of M2-associated cytokines sCD163 and IL-10 (**Figures 4G–J**).

Further, BET degradation in SUM149 cells induced broad but selective remodeling of tumor-derived cytokines associated with macrophage recruitment and polarization. Key chemotactic factors, including IL-8, were markedly reduced, suggesting impaired monocyte/macrophage recruitment. In parallel, mediators linked to M2-like TAM programming, including DKK1, lipocalin-2 and MMP9 were strongly suppressed, indicating disruption of tumor-derived pro-polarization signals. Angiogenic signaling was also attenuated, as evidenced by reduced VEGF, consistent with diminished pro-tumor TAM function.

This effect was not uniform, as a subset of inflammatory mediators, including CCL20 and CD147, was upregulated, indicating selective rewiring rather than global suppression of tumor–immune signaling. Notably, the most abundantly expressed cytokines in SUM149 cells--DKK1, IL-8, CCL20, and VEGF--were among those most significantly altered, highlighting that BET degradation reshapes dominant signaling axes within the tumor secretome. Together, these changes suggest that BET inhibition shifts the tumor secretome away from a macrophage-supportive, immunosuppressive state toward a less pro-tumorigenic and more selectively inflammatory milieu. This tumor-intrinsic rewiring is consistent with the observed phenotype-specific effects on macrophage migration and infiltration, including enhanced M1 recruitment and reduced M2 accumulation (**Supplementary Figures 7H, I**).

Consistent with these findings, STAT6, a key transcription factor driving M2 polarization, was differentially expressed across macrophage subsets, with higher total protein levels observed in M2-type macrophages compared with M0 cells. Notably, STAT6 activation, as assessed by phosphorylated STAT6 (pSTAT6), was predominantly detected in M2 macrophages. ZBC260 treatment reduced both total STAT6 expression and its activation (pSTAT6), particularly in M2 macrophages (**Figures 4K, L**).

Collectively, these results indicate that ZBC260 drives selective M1 expansion and M2 depletion through a combination of enhanced recruitment of M1 macrophages and inhibition of M2 polarization, providing a mechanistic explanation for the TAM reprogramming observed in earlier experiments.

### BETd ZBC260 enhances macrophage-mediated phagocytosis of tumor cells

**Figure 5:**
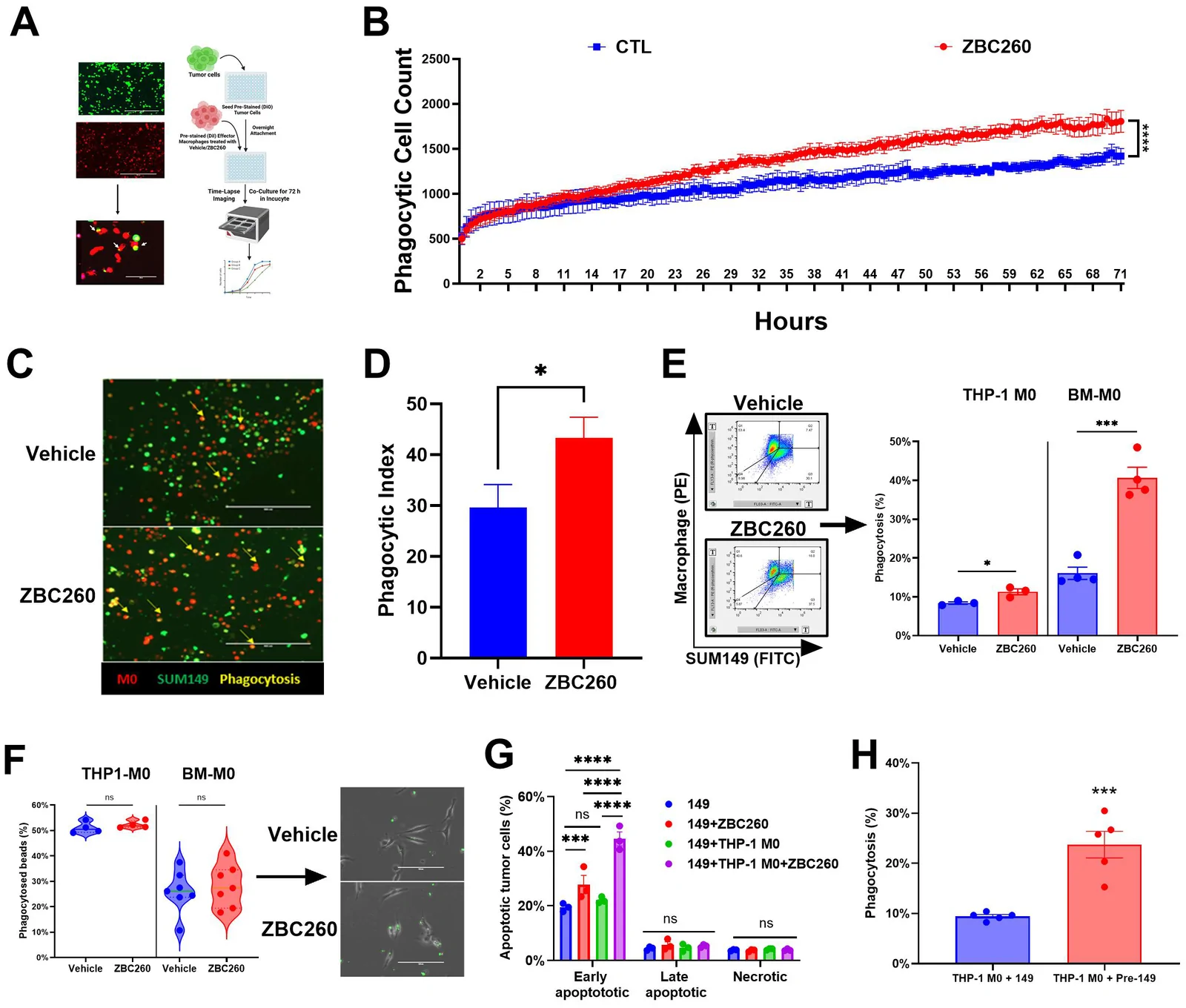
BETd ZBC260 enhances macrophage-mediated phagocytosis of tumor cells. **(A)** Schematic illustrating the evaluation of ZBC260 effect on macrophage-mediated phagocytosis of tumor cells. **(B)** Time-dependent quantification of phagocytosed SUM149 cell by THP-1-macrophages number n=5. **(C)** Representative fluorescence microscopy images showing macrophage phagocytosis of DiO-positive tumor cell following vehicle or ZBC260 treatment. (arrows; scale bar 100 μm). **(D)** Quantification of the phagocytic index, representing the percentage of DiO-positive fluorescence within macrophages, n=3. **(E)** Phagocytosis percentage after 72 hours of SUM149 and THP-1 M0 or BM-M0 co-culture quantified by flow cytometry, n=3. **(F)** Phagocytosis of opsonized beads by THP-1 M0 treated with vehicle or ZBC260, n=4 (scale bar 100 μm). **(G)** Apoptosis of SUM149 cells in co-culture with THP-1 M0 after 72 hours treatment with vehicle or ZBC260, n=3. **(H)** THP-1 M0 macrophage-mediated phagocytosis of vehicle and ZBC260 pre-treated SUM149 tumor cells analysed via flow cytometry, n=5. In (B-M), data points are the mean ± SEM for individual experiments. Data is technical and biological replicates and was analyzed by Student’s t-tests with Welch’s correction **(B, D, E, G)** and one-way ANOVA with Bonferroni’s correction **(F)**. *p<0.05; **p < 0.01; ***p < 0.001, ****p < 0.0001.

Given the increased phagosome pathways and phagocytosis score observed in myeloid cells, we next sought to determine whether ZBC260 enhances macrophage-mediated phagocytosis of tumor cells. THP-1-derived macrophages (THP-1 M0) and BM-macrophages were co-cultured with SUM149 or D2A1 tumor cells in the presence of vehicle or ZBC260. Phagocytosis was monitored over time using IncuCyte live-cell imaging. A significant increase in phagocytosis was observed beginning at ~28 hours (**Figures 5A, B**). To further assess macrophage-mediated tumor cell uptake using a complementary approach, fluorescence microscopy was performed after 72 hours of co-culture. Quantitative analysis of fluorescence microscopy images demonstrated that ZBC260 treatment significantly increased the percentage of macrophages containing DiO-positive fluorescence, indicating increased macrophage-mediated tumor cell uptake (**Figures 5C, D**). Further, flow cytometric analysis at 72 hours provided quantitative confirmation of increased phagocytosis (**Figure 5E**). Together, these complementary approaches demonstrate that ZBC260 enhances macrophage-mediated tumor cell phagocytosis. To determine whether this increase reflected intrinsic macrophage phagocytic ability or tumor-dependent effects, we assessed bead uptake in isolated macrophages. ZBC260 treatment did not significantly alter uptake of opsonized beads (**Figure 5F**), indicating that enhanced tumor phagocytosis requires tumor-derived signals or context-dependent interactions. Thus, ZBC260 does not directly increase intrinsic macrophage phagocytic capacity but may modify tumor cell properties, facilitating macrophage recognition and clearance.

Evaluation of tumor cell apoptosis after 72 hours of co-culture revealed that both ZBC260 and macrophages independently promoted tumor cell apoptosis, with an additive effect when combined (**Figure 5G**). To confirm the role of tumor priming, tumor cells were pretreated with ZBC260 and then co-cultured with macrophages. As expected, pretreated tumor cells were phagocytosed more efficiently, with increased macrophage phagocytosis observed as early as 24 hours (**Figure 5H**), supporting a tumor-dependent effect. Collectively, these findings indicate that ZBC260 enhances macrophage-mediated tumor phagocytosis in a time-dependent manner, primarily through increased tumor susceptibility and context-dependent tumor–macrophage interactions, rather than by directly enhancing intrinsic macrophage phagocytic activity.

### BETd reduces CSC frequency via macrophage-dependent mechanisms

**Figure 6:**
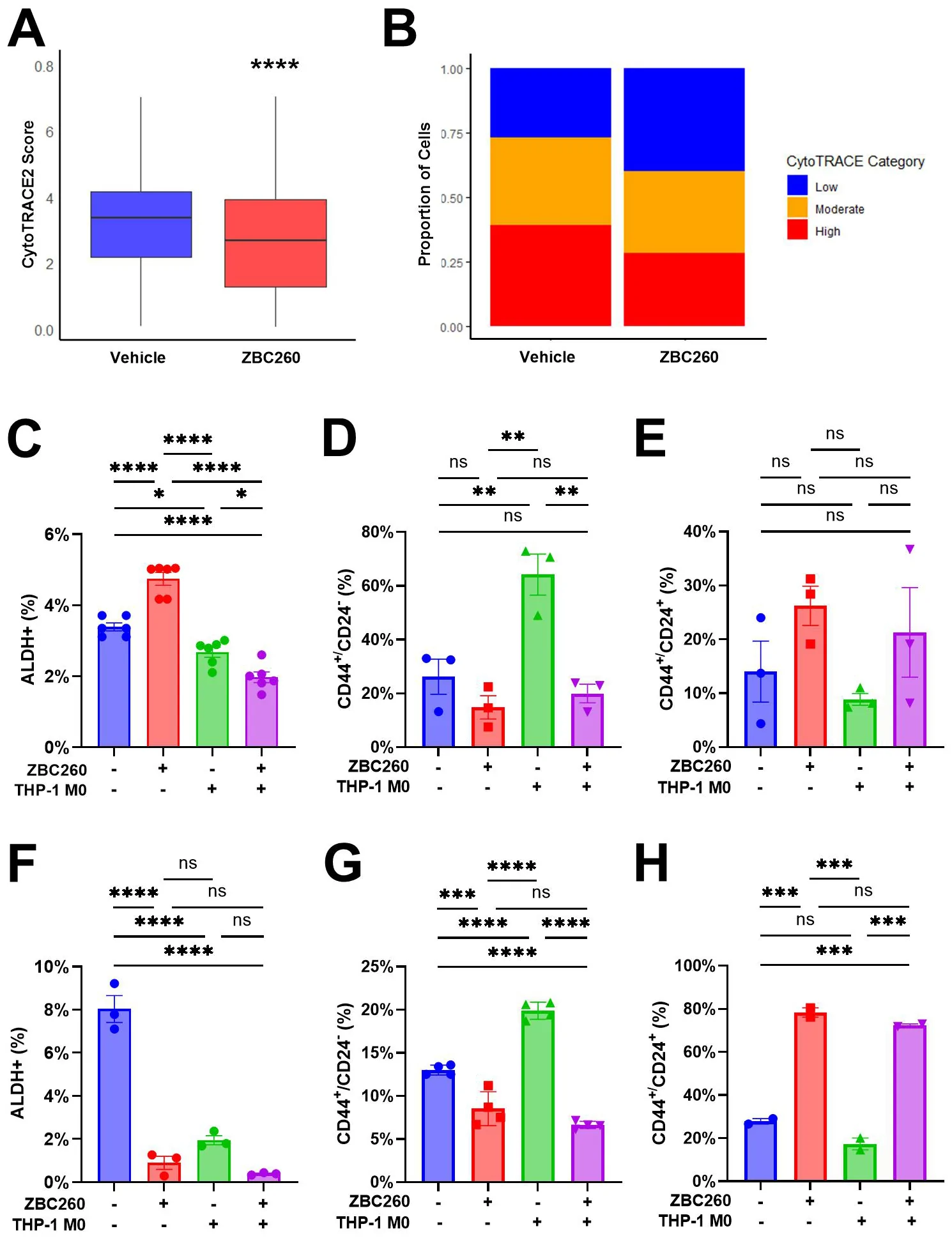
BETd reduces cancer stem cell (CSC) frequency via macrophage-dependent mechanisms. **(A)** Box plot showing CytoTRACE scores (CSC score) for vehicle and ZBC260 treated D2A1, *in vivo* tumor cells using CytoTRACE package in R, n=3. **(B)** Stacked bar chart showing the proportion of tumor cells classified as low, medium, and high CytoTRACE scores in vehicle and ZBC260 treated groups, n=3. **(C–E)** Flow cytometry evaluation of CSC markers **(C)** ALDH^+^ (n=6), **(D)** CD44^+^/CD24^-^ (n=3), and **(E)** CD44^+^/CD24^+^ (n=3) for SUM149 cells in 2D co-culture with THP-1 M0. **(F–H)** Flow cytometry evaluation of CSC markers **(F)** ALDH^+^ (n=3), **(G)** CD44^+^/CD24^-^ (n=4), and **(H)** CD44^+^/CD24^+^ (n=4) for SUM149 cells in 3D co-culture with THP-1 M0. Data is technical and biological replicates and was analyzed by Student’s t-tests with Welch’s correction **(A, B)** and one-way ANOVA with Bonferroni correction **(C–H)**. *p<0.05; **p < 0.01; ***p < 0.001, ****p < 0.0001.

We next sought to determine whether the TAM reprogramming and enhanced phagocytosis induced by ZBC260 also affect therapy-resistant CSC populations (39). To address this, we computed a CSC score from single-cell RNA-seq data of D2A1 tumors using CytoTRACE. ZBC260 treatment resulted in a significant reduction in CSC score, indicating a decrease in stem-like tumor cells (**Figure 6A**). Tumor cells were further classified into low, moderate, and high stem cell scores. Importantly, the proportion of low-score cells increased, while high-score cells were diminished, suggesting that ZBC260 promotes differentiation toward a more terminal, less stem-like state (**Figure 6B**).

To explore whether macrophages contribute to this effect, we performed co-culture experiments in which tumor cells were grown with THP-1-derived macrophages in 3D spheroid cultures and quantified spheroid formation as a measure of self-renewal capacity [9]. Our analysis showed that M0, M1, and M2-like macrophages enhanced tumorsphere formation in co-culture, an effect that was reduced following ZBC260 treatment (**Supplementary Figures 8A–C**). Because the effects were comparable regardless of macrophage polarization state, we further analyzed CSC marker expression using M0 macrophages co-cultured with tumor cells under either 2D adherent conditions (48 hours) or 3D spheroid culture conditions (14 days), with or without ZBC260 treatment (**Supplementary Figures 8D, E**).

ZBC260 treatment alone increased the percentage of ALDH^+^ cells relative to control; however, this was accompanied by a reduction in the absolute number of ALDH^+^ cells, likely reflecting an overall decrease in viable tumor cells due to cytotoxic effects. This pattern suggests a relative enrichment of the ALDH^+^ fraction among surviving cells, potentially driven by shifts in cell-state composition rather than expansion of the CSC cells (9). In contrast, macrophages alone decreased the percentage of ALDH^+^ cells while increasing total cell numbers, consistent with effects on population dynamics and possible promotion of non-CSC cell proliferation and/or differentiation (**Figure 6C**; **Supplementary Figure 8**).

Importantly, in co-culture conditions, macrophages aborgrated the ZBC260-induced increase in ALDH^+^ percentage, resulting in a further reduction in ALDH^+^ cells compared to either condition alone. This suggests that macrophage-mediated effects on cell state and differentiation can override ZBC260-associated relative enrichment of ALDH^+^ cells, while drug-induced cytotoxicity contributes to reducing the overall ALDH^+^ cell burden. Together, these findings indicate that ZBC260 and macrophages exert distinct but complementary effects on tumor cell populations, with ZBC260 primarily influencing cell survival and state distribution, and macrophages modulating CSC dynamics (**Figure 6C**; **Supplementary Figure 8C**).

Conversely, ZBC260 alone reduced both the percentage and absolute number of CD44^+^/CD24^-^ cells in 2D and 3D cultures, indicating an overall reduction in this CSC population. In contrast, macrophages alone increased the percentage of CD44^+^/CD24^-^ cells under both conditions. In 2D culture, this was accompanied by an increase in absolute cell numbers, whereas in 3D culture, the increased percentage occurred despite a reduction in absolute cell numbers, suggesting context-dependent effects of macrophages on cell survival and growth, particularly in 3D conditions.

Importantly, the combination of ZBC260 and macrophages resulted in a reduction in both the percentage and absolute number of CD44^+^/CD24^-^ cells, with a more pronounced effect observed in 3D culture. This enhanced suppression in 3D conditions is consistent with cumulative or sustained effects on cell viability, suggesting that the combination more effectively limits this CSC population over time (**Figures 6D–G**; **Supplementary Figures 8D–G**).

Furthermore, ZBC260 treatment alone increased both the percentage and absolute number of differentiated (non-CSC) CD44^+^/CD24^+^ cells in both monoculture and co-culture across 2D and 3D conditions, whereas macrophages alone had no significant effect compared to control (**Figures 6D, E**; **Supplementary Figures 8D, E**). These findings were further validated in 3D tumorsphere cultures (**Figures 6F–H**; **Supplementary Figures 8F–H**). To evaluate whether macrophage polarization differentially influences CSC activity, SUM149 cells were co-cultured with THP-1-derived M0, M1, or M2 macrophages and assessed using tumorsphere formation assays. All macrophage subsets similarly enhanced tumorsphere formation, which was consistently suppressed by ZBC260 treatment, with no significant differences observed among macrophage polarization states (**Supplementary Figures 8I–K**).

Together, these findings indicate that ZBC260 directly reduces CD44^+^/CD24^-^ CSCs, while macrophages modulate this population in a context-dependent manner, and that their combined effects lead to a more robust suppression of CSCs, particularly under long-term 3D culture conditions (**Figures 6D–G**; **Supplementary Figures 8D–G**).

**Figure 7:**
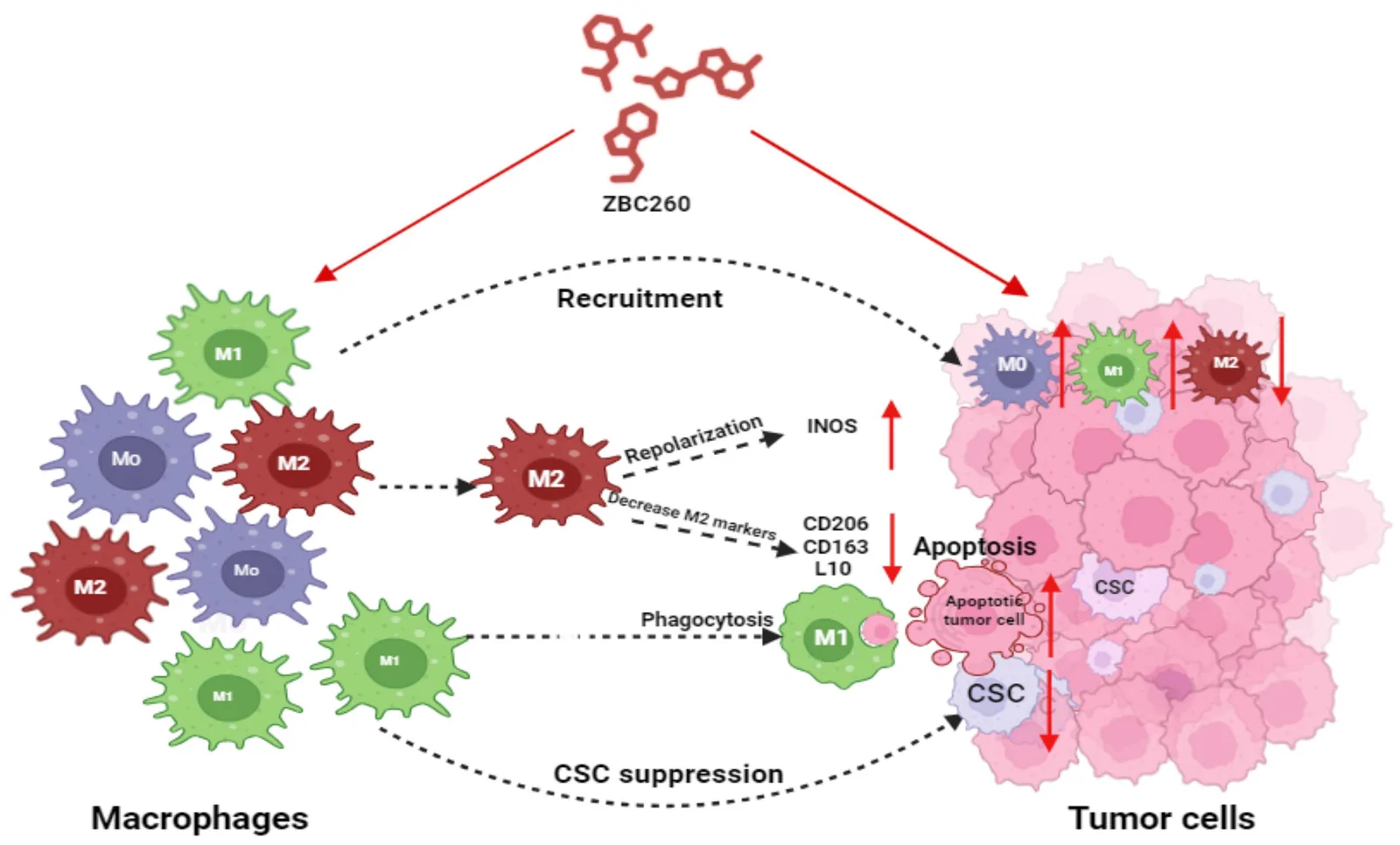
BETd inhibits breast cancer via reprograming macrophages. Graphical representation of the multilayer regulation of macrophages by BET protein degrader. Image created with BioRender.com.

Collectively, these results indicate that ZBC260 not only promotes differentiation of CSCs but also engages macrophage-dependent mechanisms to further suppress stem-like tumor populations, linking TAM reprogramming, enhanced phagocytosis, and CSC targeting into a coordinated anti-tumor program (**Figure 7**).

## Discussion

The therapeutic landscape of breast cancer continues to evolve, yet despite advances, achieving durable responses remains challenging, particularly in tumors characterized by persistent stem-like properties and an immunosuppressive TME (1, 2). Recent studies have emphasized the importance of combining approaches that directly target cancer cells with strategies that enhance anti-tumor immune responses to improve therapeutic efficacy (40, 41). In this context, our findings provide new insight into the therapeutic mechanism of the pan-BET degrader ZBC260 by demonstrating that its anti-tumor activity involves coordinated suppression of CSC-associated phenotypes and remodeling of immune cell function. In this study, we demonstrate that BETd reprograms TME cells to enhance anti-tumor immunity, reprogram macrophage function, and suppress CSC-associated properties in breast cancer. While BRD4 inhibition has been shown to activate NK cells (42), the anti-tumor efficacy of the pan-BET degrader ZBC260 appears to be independent of NK cells. Instead, BETd ZBC260 effects are mediated primarily by macrophages, consistent with prior work showing that BET targeting promotes anti-tumor responses via modulation of macrophage function and polarization (43). Using the D2A1 immunocompetent murine model, we present the first evidence that depletion of macrophages *in vivo* significantly diminishes the anti-tumor and anti-stemness effects of BETd, establishing macrophages as critical mediators of the therapeutic efficacy of this compound. Therefore, the current work focuses on elucidating macrophage-dependent mechanisms underlying the therapeutic effects of ZBC260.

Macrophages display high phenotypic plasticity, responding to microenvironmental cues to polarize into distinct functional subtypes (18, 44). In alignment with previous research (45, 46), our scRNA-seq analysis identified multiple TAM subsets in breast tumors, including immunosuppressive Mrc1^+^, and Arg1^+^ TAMs linked to poor prognosis (47) and antigen-presenting, inflammatory Cd74^+^ TAMs associated with improved survival (48). These observations are consistent with recent single-cell and spatial transcriptomic studies that reveal TAMs exist along a functional continuum and display context-dependent transcriptional programs influencing tumor progression and therapy response in solid tumors (23, 49–51). Treatment with BETd reshaped the distribution of TAM functional states, reducing immunosuppressive Mrc1+TAMs while enhancing antigen-presenting Cd74+TAMs (49).

Furthermore, BETd selectively decreased M2-type macrophage recruitment to tumorspheres while promoting M1-type migration, potentially through distinct signaling pathways (52). These findings underscore the dual role of BETd in reprogramming TAM phenotypes and differentially regulating their recruitment, demonstrating that BETd treatment reshapes the immune landscape of the TME.

TF analysis identified *Stat1, and Jun* as pivotal regulators mediating the effects of BETd on macrophages. The Stat1 TF, a well-established driver of M1-type macrophage polarization (53) was upregulated in response to BETd in myeloid cells. Correlation analysis demonstrated a strong association between Stat1 expression and the expression of Gbp2b and Fgl2, both of which are implicated in M1-type polarization (54, 55). This upregulation corresponds with the increased prevalence of M1-type TAMs, and the elevated expression of M1-type markers and cytokines observed in both THP-1 and murine macrophages (30). In contrast, STAT6, a critical regulator of M2-type polarization (53), exhibited reduced protein expression and activation after BETd treatment in THP-1-macrophages. This reduction aligns with the downregulation of M2-type markers and cytokines (32).

GO analysis indicated an upregulation of pathways involved in innate immune response and leukocyte migration, and enrichment analysis via KEGG further highlighted enhanced activity in antigen processing and presentation, phagosome formation, and efferocytosis. These findings indicate that BETd treatment augments macrophage-mediated tumor clearance by promoting both phagocytic activity and tumor antigen presentation. Consistent with this, we observed increased phagocytosis scores, upregulation of multiple MHC-I genes (H2-K1, H2-D1, H2-EB1, H2-AB1, B2M). BETd also enhanced expression of cytokines that recruit T cells, correlating with increased lymphoid infiltration in D2A1 tumors. In contrast, loss of Brd7 has been reported to suppress MHC-I expression and interferon signaling, reducing tumor immunogenicity (56), suggesting that different bromodomain-containing proteins may have opposing effects on immune recognition. Our findings are consistent with previous studies suggesting that enhanced phagocytosis can improve antigen cross-presentation, effectively bridging innate and adaptive immune responses (57). Additionally, *in vitro* co-culture experiments demonstrated that BETd augments macrophage-mediated phagocytosis of tumor cells independently of Fc receptor-mediated pathways (58). These findings align with previous research showing that tumor–macrophage interactions drive phagocytosis and underscore the critical role of TAM phagocytic activity in controlling tumor progression (59).

To evaluate the functional impact of these altered interactions on cancer stemness, we calculated stemness scores and performed limiting dilution reimplantation assays. These assays showed a significant reduction in CSC populations within the TME, which was attenuated by macrophage depletion. Consistently, our *in vitro* data indicate that ZBC260 and macrophages regulate tumor cell plasticity through distinct and context-dependent mechanisms. ZBC260 reduces the CD44^+^/CD24^-^ CSC subpopulation in both 2D and 3D cultures, indicating a direct suppressive effect on stem-like cells. In 2D culture, the increase in ALDH^+^ percentage despite reduced absolute numbers in contrast to long term 3D culture suggests a selective survival or state-shifting effect among remaining cells, which is consistent with our previous findings that ZBC260 promotes differentiation-like transitions within the tumor cell population (9). In contrast, macrophages reshape tumor heterogeneity by increasing total cell numbers while reducing the relative ALDH^+^ fraction, suggesting that they may preferentially support expansion of non-CSC-like cells or promote differentiation away from ALDH^+^ states, thereby altering population balance rather than directly expanding CSCs. In contrast, macrophage co-culture promotes expansion of the CD44^+^/CD24^-^ CSC fraction, consistent with previous reports (60), supporting a macrophage-driven niche that sustains or enhances tumor cell plasticity. This is accompanied by altered ALDH^+^ distribution and changes in total cell numbers, indicating that macrophages modulate tumor population structure in a context and marker-dependent manner. Importantly, in co-culture, macrophages counteract the ZBC260-associated enrichment of ALDH^+^ cells and further enhance suppression of the CD44^+^/CD24^-^ population, particularly in 3D culture conditions. This indicates that macrophage-mediated remodeling of cell state can dominate ZBC260-induced selection effects. Together, these results support a model in which ZBC260 promotes CSC depletion and differentiation bias, while macrophages actively reprogram tumor cell state distribution, with their combined action producing a stronger and more sustained suppression of stem-like populations.

TAMs are known to sustain CSC populations by modulating CSC-specific signaling pathways (4, 60). The observed inhibition of CSCs with BETd likely arises from a dual mechanism: 1) targeting of CSC-associated inflammatory pathways, as previously described (9), and 2) reprogramming of TAM phenotype and function, as shown in this study.

Our findings demonstrate the BET degrader ZBC260 exerts significant anti-tumor and anti-stemness effects by reprogramming macrophage phenotypes, selectively modulating recruitment and enhancing macrophage tumoricidal activity. These results highlight the important role of BET proteins in the TME and underscore the therapeutic potential of BET degradation to leverage macrophage-mediated anti-tumor immunity, offering a compelling strategy to reshape the tumor immune landscape, reduce CSC populations, and inhibit tumor progression. While the current study focused on primary tumor growth and macrophage-mediated mechanisms, future investigations will evaluate the impact of BET degradation on tumor microenvironment and its role in tumor growth, metastatic progression, including bone dissemination.

## Data Availability

The original contributions presented in the study are publicly available. This data can be found here: NCBI GEO, accession number GSE345296.

## References

[1] Lukasiewicz S Czeczelewski M Forma A Baj J Sitarz R Stanislawek A . Breast cancer-epidemiology, risk factors, classification, prognostic markers, and current treatment strategies-an updated review. Cancers (Basel). (2021) 13(17):4287. doi: 10.3390/cancers13174287 34503097 PMC8428369

[2] Barzaman K Karami J Zarei Z Hosseinzadeh A Kazemi MH Moradi-Kalbolandi S . Breast cancer: biology, biomarkers, and treatments. Int Immunopharmacol. (2020) 84:106535. doi: 10.1016/j.intimp.2020.106535 32361569

[3] Ribeiro R Carvalho MJ Goncalves J Moreira JN . Immunotherapy in triple-negative breast cancer: insights into tumor immune landscape and therapeutic opportunities. Front Mol Biosci. (2022) 9:903065. doi: 10.3389/fmolb.2022.903065 36060249 PMC9437219

[4] Allavena P Digifico E Belgiovine C . Macrophages and cancer stem cells: a malevolent alliance. Mol Med. (2021) 27:121. doi: 10.1186/s10020-021-00383-3 34583655 PMC8480058

[5] Bian J Fu J Wang X Lee J Brar G Escorcia FE . Characterization of immunogenicity of Malignant cells with stemness in intrahepatic cholangiocarcinoma by single-cell RNA sequencing. Stem Cells Int. (2022) 2022:3558200. doi: 10.1155/2022/3558200 35530414 PMC9076354

[6] Al-Hajj M Wicha MS Benito-Hernandez A Morrison SJ Clarke MF . Prospective identification of tumorigenic breast cancer cells. Proc Natl Acad Sci USA. (2003) 100:3983–8. doi: 10.1073/pnas.0530291100 12629218 PMC153034

[7] Sarnik J Poplawski T Tokarz P . BET proteins as attractive targets for cancer therapeutics. Int J Mol Sci. (2021) 22:11102. doi: 10.3390/ijms222011102 34681760 PMC8538173

[8] Xavier PLP Cordeiro YG Alexandre PA Pires PRL Saranholi BH Silva ER . An epigenetic screening determines BET proteins as targets to suppress self-renewal and tumorigenicity in canine mammary cancer cells. Sci Rep. (2019) 9:17363. doi: 10.1038/s41598-019-53915-7 31758045 PMC6874531

[9] Sharma D Hager CG Shang L Tran L Zhu Y Ma A . The BET degrader ZBC260 suppresses stemness and tumorigenesis and promotes differentiation in triple-negative breast cancer by disrupting inflammatory signaling. Breast Cancer Res. (2023) 25:144. doi: 10.1186/s13058-023-01715-3 37968653 PMC10648675

[10] da Motta LL Ledaki I Purshouse K Haider S De Bastiani MA Baban D . The BET inhibitor JQ1 selectively impairs tumour response to hypoxia and downregulates CA9 and angiogenesis in triple negative breast cancer. Oncogene. (2017) 36:122–32. doi: 10.1038/onc.2016.184 27292261 PMC5061082

[11] Miao Y Yang H Levorse J Yuan S Polak L Sribour M . Adaptive immune resistance emerges from tumor-initiating stem cells. Cell. (2019) 177:1172–86:e14. doi: 10.1016/j.cell.2019.03.025 31031009 PMC6525024

[12] Choi SK Hong SH Kim HS Shin CY Nam SW Choi WS . JQ1, an inhibitor of the epigenetic reader BRD4, suppresses the bidirectional MYC-AP4 axis via multiple mechanisms. Oncol Rep. (2016) 35:1186–94. doi: 10.3892/or.2015.4410 26573731

[13] Abruzzese MP Bilotta MT Fionda C Zingoni A Soriani A Vulpis E . Inhibition of bromodomain and extra-terminal (BET) proteins increases NKG2D ligand MICA expression and sensitivity to NK cell-mediated cytotoxicity in multiple myeloma cells: role of cMYC-IRF4-miR-125b interplay. J Hematol Oncol. (2016) 9:134. doi: 10.1186/s13045-016-0362-2 27903272 PMC5131470

[14] Wu Y Jennings NB Sun Y Dasari SK Bayraktar E Corvigno S . Targeting CCR2(+) macrophages with BET inhibitor overcomes adaptive resistance to anti-VEGF therapy in ovarian cancer. J Cancer Res Clin Oncol. (2022) 148:803–21. doi: 10.1007/s00432-021-03885-z 35094142 PMC8930900

[15] Zhang Z Zhang Q Xie J Zhong Z Deng C . Enzyme-responsive micellar JQ1 induces enhanced BET protein inhibition and immunotherapy of Malignant tumors. Biomater Sci. (2021) 9:6915–26. doi: 10.1039/d1bm00724f 34524279

[16] Zhou B Hu J Xu F Chen Z Bai L Fernandez-Salas E . Discovery of a small-molecule degrader of bromodomain and extra-terminal (BET) proteins with picomolar cellular potencies and capable of achieving tumor regression. J Med Chem. (2018) 61:462–81. doi: 10.1021/acs.jmedchem.6b01816 28339196 PMC5788414

[17] Tian T Guo T Zhen W Zou J Li F . BET degrader inhibits tumor progression and stem-like cell growth via Wnt/beta-catenin signaling repression in glioma cells. Cell Death Dis. (2020) 11:900. doi: 10.1038/s41419-020-03117-1 33093476 PMC7582157

[18] Qiu X Zhao T Luo R Qiu R Li Z . Tumor-associated macrophages: key players in triple-negative breast cancer. Front Oncol. (2022) 12:772615. doi: 10.3389/fonc.2022.772615 35237507 PMC8882594

[19] Correia AL Guimaraes JC Auf der Maur P De Silva D Trefny MP Okamoto R . Author correction: hepatic stellate cells suppress NK cell-sustained breast cancer dormancy. Nature. (2021) 600:E7. doi: 10.1038/s41586-021-04104-y 34764479

[20] Bushnell GG Sharma D Wilmot HC Zheng M Fashina TD Hutchens CM . Natural killer cell regulation of breast cancer stem cells mediates metastatic dormancy. Cancer Res. (2024) 84:3337–53. doi: 10.1158/0008-5472.can-24-0030 39106452 PMC11474167

[21] Murray PJ . Macrophage polarization. Annu Rev Physiol. (2017) 79:541–66. doi: 10.1146/annurev-physiol-022516-034339 27813830

[22] Guan F Wang R Yi Z Luo P Liu W Xie Y . Tissue macrophages: origin, heterogenity, biological functions, diseases and therapeutic targets. Signal Transduct Target Ther. (2025) 10:93. doi: 10.1038/s41392-025-02124-y 40055311 PMC11889221

[23] Rakina M Larionova I Kzhyshkowska J . Macrophage diversity in human cancers: new insight provided by single-cell resolution and spatial context. Heliyon. (2024) 10:e28332. doi: 10.1016/j.heliyon.2024.e28332 38571605 PMC10988020

[24] Zhang Y Zhong F Liu L . Single-cell transcriptional atlas of tumor-associated macrophages in breast cancer. Breast Cancer Res. (2024) 26:129. doi: 10.1186/s13058-024-01887-6 39232806 PMC11373130

[25] Nguyen TDT Lee AJ Park HJ Shah N Mandzhieva B Lee DS . Pan-cancer single-cell RNA sequencing analysis refines multi-origin monocyte and macrophage lineages. Cancer Immunol Res. (2026) 14:350–66. doi: 10.21203/rs.3.rs-3229780/v1 41231218 PMC12865363

[26] Ye Y Ma J Zhang Q Xiong K Zhang Z Chen C . A CTL/M2 macrophage-related four-gene signature predicting metastasis-free survival in triple-negative breast cancer treated with adjuvant radiotherapy. Breast Cancer Res Treat. (2021) 190:329–41. doi: 10.21203/rs.3.rs-527123/v1 34482483

[27] Weng YS Tseng HY Chen YA Shen PC Al Haq AT Chen LM . MCT-1/miR-34a/IL-6/IL-6R signaling axis promotes EMT progression, cancer stemness and M2 macrophage polarization in triple-negative breast cancer. Mol Cancer. (2019) 18:42. doi: 10.1186/s12943-019-0988-0 30885232 PMC6421700

[28] DeNardo DG Ruffell B . Macrophages as regulators of tumour immunity and immunotherapy. Nat Rev Immunol. (2019) 19:369–82. doi: 10.1038/s41577-019-0127-6 30718830 PMC7339861

[29] Su P Li O Ke K Jiang Z Wu J Wang Y . Targeting tumor-associated macrophages: critical players in tumor progression and therapeutic strategies (review). Int J Oncol. (2024) 64:60. doi: 10.3892/ijo.2024.5648 38695252 PMC11087038

[30] Genin M Clement F Fattaccioli A Raes M Michiels C . M1 and M2 macrophages derived from THP-1 cells differentially modulate the response of cancer cells to etoposide. BMC Cancer. (2015) 15:577. doi: 10.1186/s12885-015-1546-9 26253167 PMC4545815

[31] Pineda-Torra I Gage M de Juan A Pello OM . Isolation, culture, and polarization of murine bone marrow-derived and peritoneal macrophages. Methods Mol Biol. (2015) 1339:101–9. doi: 10.1007/978-1-4939-2929-0_6 26445783

[32] Nasser H Adhikary P Abdel-Daim A Noyori O Panaampon J Kariya R . Establishment of bone marrow-derived M-CSF receptor-dependent self-renewing macrophages. Cell Death Discov. (2020) 6:63. doi: 10.1038/s41420-020-00300-3 32714570 PMC7378060

[33] Satija R Farrell JA Gennert D Schier AF Regev A . Spatial reconstruction of single-cell gene expression data. Nat Biotechnol. (2015) 33:495–502. doi: 10.1038/nbt.3192 25867923 PMC4430369

[34] Li K Sun L Wang Y Cen Y Zhao J Liao Q . Single-cell characterization of macrophages in uveal melanoma uncovers transcriptionally heterogeneous subsets conferring poor prognosis and aggressive behavior. Exp Mol Med. (2023) 55:2433–44. doi: 10.1038/s12276-023-01115-9 37907747 PMC10689813

[35] Chen J Liao S Xiao Z Pan Q Wang X Shen K . The development and improvement of immunodeficient mice and humanized immune system mouse models. Front Immunol. (2022) 13:1007579. doi: 10.3389/fimmu.2022.1007579 36341323 PMC9626807

[36] Morris VL Tuck AB Wilson SM Percy D Chambers AF . Tumor progression and metastasis in murine D2 hyperplastic alveolar nodule mammary tumor cell lines. Clin Exp Metastasis. (1993) 11:103–12. doi: 10.1007/bf00880071 8422701

[37] Liu L Ye Y Zhu X . MMP-9 secreted by tumor associated macrophages promoted gastric cancer metastasis through a PI3K/AKT/Snail pathway. BioMed Pharmacother. (2019) 117:109096. doi: 10.1016/j.biopha.2019.109096 31202170

[38] Wei C Ma Y Wang M Wang S Yu W Dong S . Tumor-associated macrophage clusters linked to immunotherapy in a pan-cancer census. NPJ Precis Oncol. (2024) 8:176. doi: 10.1038/s41698-024-00660-4 39117688 PMC11310399

[39] Codony-Servat J Rosell R . Cancer stem cells and immunoresistance: clinical implications and solutions. Transl Lung Cancer Res. (2015) 4(6):689–703. doi: 10.3978/j.issn.2218-6751.2015.12.11 26798578 PMC4700228

[40] Chen DS Mellman I . Elements of cancer immunity and the cancer-immune set point. Nature. (2017) 541:321–30. doi: 10.1038/nature21349 28102259

[41] Binnewies M Roberts EW Kersten K Chan V Fearon DF Merad M . Understanding the tumor immune microenvironment (TIME) for effective therapy. Nat Med. (2018) 24:541–50. doi: 10.1038/s41591-018-0014-x 29686425 PMC5998822

[42] Reggiani F Talarico G Gobbi G Sauta E Torricelli F Manicardi V . BET inhibitors drive natural killer activation in non-small cell lung cancer via BRD4 and SMAD3. Nat Commun. (2024) 15:2567. doi: 10.1038/s41467-024-46778-8 38519469 PMC10960013

[43] Li X Fu Y Yang B Guo E Wu Y Huang J . BRD4 inhibition by AZD5153 promotes antitumor immunity via depolarizing M2 macrophages. Front Immunol. (2020) 11:89. doi: 10.3389/fimmu.2020.00089 32184777 PMC7058627

[44] Mills CD . Anatomy of a discovery: m1 and m2 macrophages. Front Immunol. (2015) 6:212. doi: 10.3389/fimmu.2015.00212 25999950 PMC4419847

[45] Qian J Olbrecht S Boeckx B Vos H Laoui D Etlioglu E . A pan-cancer blueprint of the heterogeneous tumor microenvironment revealed by single-cell profiling. Cell Res. (2020) 30:745–62. doi: 10.1038/s41422-020-0355-0 32561858 PMC7608385

[46] Xu L Chen Y Liu L Hu X He C Zhou Y . Tumor-associated macrophage subtypes on cancer immunity along with prognostic analysis and SPP1-mediated interactions between tumor cells and macrophages. PloS Genet. (2024) 20:e1011235. doi: 10.1371/journal.pgen.1011235 38648200 PMC11034676

[47] Haque A Moriyama M Kubota K Ishiguro N Sakamoto M Chinju A . CD206(+) tumor-associated macrophages promote proliferation and invasion in oral squamous cell carcinoma via EGF production. Sci Rep. (2019) 9:14611. doi: 10.1038/s41598-019-51149-1 31601953 PMC6787225

[48] Cheng J Li J Jiang X Ma X Li B Zhai H . CD74 facilitates immunotherapy response by shaping the tumor microenvironment of hepatocellular carcinoma. Mol Med. (2024) 30:116. doi: 10.1186/s10020-024-00884-x 39118044 PMC11308498

[49] Zhang L Li Z Skrzypczynska KM Fang Q Zhang W O'Brien SA . Single-cell analyses inform mechanisms of myeloid-targeted therapies in colon cancer. Cell. (2020) 181:442–59:e29. doi: 10.1016/j.cell.2020.03.048 32302573

[50] Laviron M Petit M Weber-Delacroix E Combes AJ Arkal AR Barthelemy S . Tumor-associated macrophage heterogeneity is driven by tissue territories in breast cancer. Cell Rep. (2022) 39:110865. doi: 10.1016/j.celrep.2022.110865 35613577

[51] Mei S Zhang H Hirz T Jeffries NE Xu Y Baryawno N . Single-cell and spatial transcriptomics reveal a tumor-associated macrophage subpopulation that mediates prostate cancer progression and metastasis. Mol Cancer Res. (2025) 23:653–65. doi: 10.1158/1541-7786.mcr-24-0791 40105746 PMC12221797

[52] Cui K Ardell CL Podolnikova NP Yakubenko VP . Distinct migratory properties of M1, M2, and resident macrophages are regulated by alpha(D)beta(2) and alpha(M)beta(2) integrin-mediated adhesion. Front Immunol. (2018) 9:2650. doi: 10.3389/fimmu.2018.02650 30524429 PMC6262406

[53] Liu T Li Y Xu M Huang H Luo Y . PRMT2 silencing regulates macrophage polarization through activation of STAT1 or inhibition of STAT6. BMC Immunol. (2024) 25:1. doi: 10.1186/s12865-023-00593-w 38172698 PMC10765854

[54] Li X Liu J Zeng M Yang K Zhang S Liu Y . GBP2 promotes M1 macrophage polarization by activating the notch1 signaling pathway in diabetic nephropathy. Front Immunol. (2023) 14:1127612. doi: 10.3389/fimmu.2023.1127612 37622120 PMC10445759

[55] Tao R Han M Yuan W Xiao F Huang J Wang X . Fibrinogen-like protein 2 promotes proinflammatory macrophage polarization and mitochondrial dysfunction in liver fibrosis. Int Immunopharmacol. (2023) 117:109631. doi: 10.1016/j.intimp.2022.109631 36878044

[56] Mondal J Zhang J Qing F Li S Kumar D Huse JT . Brd7 loss reawakens dormant metastasis initiating cells in lung by forging an immunosuppressive niche. Nat Commun. (2025) 16:1378. doi: 10.1038/s41467-025-56347-2 39910049 PMC11799300

[57] von Roemeling CA Wang Y Qie Y Yuan H Zhao H Liu X . Therapeutic modulation of phagocytosis in glioblastoma can activate both innate and adaptive antitumour immunity. Nat Commun. (2020) 11:1508. doi: 10.1038/s41467-020-15129-8 32198351 PMC7083893

[58] Nimmerjahn F Ravetch JV . Fcgamma receptors as regulators of immune responses. Nat Rev Immunol. (2008) 8:34–47. doi: 10.1038/nri2206 18064051

[59] Lecoultre M Dutoit V Walker PR . Phagocytic function of tumor-associated macrophages as a key determinant of tumor progression control: a review. J Immunother Cancer. (2020) 8:e001408. doi: 10.1136/jitc-2020-001408 33335026 PMC7747550

[60] Wang L Zhang L Zhao L Shao S Ning Q Jing X . VEGFA/NRP-1/GAPVD1 axis promotes progression and cancer stemness of triple-negative breast cancer by enhancing tumor cell-macrophage crosstalk. Int J Biol Sci. (2024) 20:446–63. doi: 10.7150/ijbs.86085 38169627 PMC10758102

