# Supplementary material for: Targeting bromodomain and extra-terminal proteins reprograms macrophages and inhibits breast cancer progression
Source: Front Immunol. 2026 Sep 3;17:1738705. doi: 10.3389/fimmu.2026.1738705 (PMC13582568; doi:10.3389/fimmu.2026.1738705)
Supplement: Supplementary file 1 [file Table1.pdf]

Supplementary Tables

Antibodies	Catalog# and Companies	RRID
**Flow Cytometry**		
CD24	Cat# 555427, BioLegend	AB_395821
CD44	Cat# 555482, BioLegend	AB_2737962
CD45	Cat#103128, BioLegend	AB_493715
CD49b	Cat#108908, BioLegend	AB_313415
F4/80	Cat#123114, BioLegend	AB_893478
**Multiplex Immunofluorescence Staining**		
Arginase-1 (Opal 690)	Cat#93668, Cell Signaling Technology	AB_2800207
CD45 (Opal 480)	Cat#70257, Cell Signaling Technology	AB_2799780
CD49b (Opal 570)	Cat#Ab181548, ABCAM	AB_2847852
F4/80 (Opal 520)	Cat#70076, Cell Signaling Technology	AB_2799771
Ly6G (Opal 620)	Cat#Ab238132, ABCAM	AB_2923218
Pan-Cytokeratin (Opal 780)	Cat#Ab9377, ABCAM	AB_307222
Ventana Omnimap anti-rabbit HRP	Cat# 760-4311, Roshe Diagnostics	AB_2811043
**Western Blot**		
BRD2	Cat#Ab243865, ABCAM	AB_3694209
BRD3	Cat#Ab264420, ABCAM	AB_3068543
BRD4	Cat#A301985A100, BETHYL	AB_2620184
β-Actin	Cat#A3854, Sigma-Aldrich	AB_262011
Stat6	Cat#5397, Cell Signaling Technology	AB_11220421
pStat6	Cat#56554, Cell Signaling Technology	AB_2799514
Secondary anti-rabbit-HRP	Cat#7074, Cell Signaling Technology	AB_2099233

Supplementary Table 1: Antibodies used for flow cytometry, multiplex immunofluorescence staining, and western blots.
