# Supplementary material for: Targeting bromodomain and extra-terminal proteins reprograms macrophages and inhibits breast cancer progression
Source: Front Immunol. 2026 Sep 3;17:1738705. doi: 10.3389/fimmu.2026.1738705 (PMC13582568; doi:10.3389/fimmu.2026.1738705)
Supplement: Supplementary file 2 [file Table2.pdf]

Genes	Probe number
GAPDH	Hs02786624_g1
CD80	Hs01045161_m1
CD163	Hs00174705_m1
CD206	Hs00267207_m1
IL1B	Hs01555410_m1
IL10	Hs00961622_m1
INOS	Hs01075529_m1
Cd86	Mm00444540_m1
Nos2	Mm00440502_m1
Mrc1	Mm01329362_m1

Supplementary Table 2: Human TaqMan Gene Expression Assay primer/probes (ThermoFisher Scientific) used for real-time quantitative PCR.
