# Supplementary material for: Targeting bromodomain and extra-terminal proteins reprograms macrophages and inhibits breast cancer progression
Source: Front Immunol. 2026 Sep 3;17:1738705. doi: 10.3389/fimmu.2026.1738705 (PMC13582568; doi:10.3389/fimmu.2026.1738705)
Supplement: Supplementary file 3 [file DataSheet1.docx]

**Supplementary Figures**

**Supplementary Figure 1: Effect of ZBC260 on BET proteins expression, D2A1 cell viability measured by MTT assay, and tumorsphere formation efficiency. (A)** Representatives immunoblot of lysate from D2A1 cells treated with ZBC260 for 72 hours and **(B)** Quantification of BRD2, BRD3, and BRD4 protein expression without (blue) and with 25nm ZBC260 (red). Protein expression was normalized to actin, n=3. **(C)** Effect of ZBC260 treatment on the D2A1 cell viability after 72 hours of treatment, n=12. **(D)** Effect of ZBC260 treatment on the D2A1 cell tumorsphere formation ability, n=6. Data points are the mean ±SEM for individual experiments. Data was analyzed by Student’s t-tests with Welch’s correction and one-way ANOVA with Bonferroni’s correction. *P< 0.05; **P < 0.01; ***P < 0.001, ****P<0.0001.

**Supplementary Figure 2: Effect of ZBC260 on NK-92MI cells, and validation of NK and macrophage cell depletion in mice. (A)** Gating scheme and quantification of NK cells (% CD49b^+^ of CD45^+^ cells) in control mice vs Anti-ASGM1 NK-depleted mice in **(B)** spleen, n=3 **(C)** and bone marrow, n=3. **(D)** Representatives immunoblot of lysate from NK92MI cells treated with ZBC260 and **(E)** Quantification of BRD2, BRD3, and BRD4 protein expression. Protein expression was normalized to Actin, n=3. **(F)** Effect of ZBC260 treatment on the NK-92MI cell viability, n=14. **(G)** Percentage of NK cell cytotoxicity after 24 hours of tumor and NK cell co-culture with vehicle or ZBC260, n=13. **(H)** Colony formation efficiency, reported as number of colonies, of tumor cells co-cultured with NK cells in the presence of vehicle or ZBC260, n=6. **(I)** Tumorsphere number of tumor cells co-cultured with NK cells in the presence of vehicle or ZBC260, n=6. **(J)** Percentage of NK cell migration towards vehicle or ZBC260 treated tumor-conditioned media and representative images of migrated NK cells, n=12. Cell percentage was calculated compared to media. **(K-M)** Quantification of macrophages (% F4/80+ of CD45^+^ cells) in Clodrosome treated mice compare to control (L) spleen, n=3 (M) and bone marrow, n=3. **(N)** Pairwise comparison of TIC frequency between groups using Pr(>Chisq) to identify the significance between each experimental group in the ELDA (Figure 1F). **(O)** P.value representing the overall likelihood ratio for differences in TIC frequency across all groups in the ELDA (Figure 1F). Data points are the mean ±SEM for individual experiments. Data was analyzed by Student’s t-tests with Welch’s correction and one-way ANOVA with Bonferroni’s correction. *P< 0.05; **P < 0.01; ***P < 0.001, ****P<0.0001.

**Supplementary Figure 3: Characterization of major subcluster cell types in scRNA-seq data. (A–C)** scRNA-seq analysis was performed on in vivo tumor tissue from D2A1 tumor-bearing mice treated with vehicle or ZBC260 (n=3). (A) UMAP plot showing the clustering of major cell types in the scRNA-seq data. Each point represents a single cell, colored by its assigned major cell type. (B) Dot plot showing the expression of unbiased marker genes for each major cell cluster. The size of the dots represents the percentage of cells expressing each marker, while the color indicates the average expression level. (C) Feature plot showing the expression of three selected genes for each major cell cluster on the UMAP.  Data points are the mean ±SEM for individual experiments. **(D)** Effect of ZBC260 treatment on the proportions of Lymphoid, Endothelial, Fibroblast, and Myeloid cell populations.

**Supplementary Figure 4: Expression of biased markers in myeloid cells. (A–E)** scRNA-seq analysis (n=3). **(A)** Feature plots showing markers of myeloid cell subpopulations. **(B)** Stacked violin plot showing the expression of myeloid cell markers across the clusters. **(C)** Dot plot illustrating cell cycle marker expression across myeloid subpopulations. **(D)** Heatmap visualizing the expression of heat shock proteins in myeloid subpopulations.**(E)** Density plots depicting the expression of M1-type TAMs markers on myeloid populations.  **(F)** Dot plot showing neutrophils markers across myeloid subpopulations.

**Supplementary Figure 5: Pathway analysis in myeloid subpopulations (A)** KEGG analysis identifying the top enriched significant (p<0.05) pathways in each myeloid subpopulation, n=3. **(B)** Gene Ontology analysis highlighting the top biological pathways in each myeloid subpopulation, with color indicating the log₁₀(P) value n=3. **(C)** Bubble plot showing the top ligand-receptor interaction pairs between myeloid and tumor cells.

**Supplementary Figure 6: Analysis of biological pathways and gene expression changes in TAMS after ZBC260 treatment. (A–F)** scRNA-seq analysis (n=3) **(A)** Gene Ontology analysis showing the top downregulated biological pathways in TAMS after ZBC260 treatment. Dot color represents log₁₀(P) value, and dot size indicates gene counts. **(B)** Significantly downregulated pathways in TAMS after ZBC260 treatment, analyzed by KEGG. **(C)** Heatmap showing the top 20 differentially expressed genes (DEGs) across myeloid clusters. **(D)** Feature plot showing phagocytosis in vehicle and ZBC260 treated myeloid cells. **(E)** Feature plot showing M1 polarization score in vehicle and ZBC260 treated myeloid cells. **(F)** Feature plot showing M2 polarization score in vehicle and ZBC260 treated myeloid cells.

**Supplementary Figure 7: Effect of ZBC260 on THP1 and murine macrophages. (A)** Schematic representation of THP1 monocyte differentiation into macrophages, followed by polarization into M1 and M2 macrophage subtypes. **(B)** Effect of ZBC260 on cell viability in different subtypes of THP1 macrophages, n=3. **(C)** Effect of ZBC260 on cell viability in BM M0, M1, and M2 macrophages **(D)** Representative Western blot showing the effect of ZBC260 on BET proteins and actin in THP1 macrophage subtypes. **(E)** Quantification of BET proteins relative to Actin. **(F)** Expression of M1 and M2 polarization markers in THP1 (M0, M1, and M2) **(G)** Expression of M1 and M2 polarization markers in BM (M0, M1, and M2). **(H)** Cytokine expression in SUM149 cells treated with vehicle or ZBC260 (25nM), quantified as integrated pixel density of cytokine array blots. **(I)** Representative cytokine array blots from SUM149 cells treated with vehicle or ZBC260 (25nM). Data was analyzed by Student’s t-tests with Welch’s correction and one-way ANOVA with Bonferroni’s correction. *P< 0.05; **P < 0.01; ***P < 0.001, ****P<0.0001.

**Supplementary Figure 8: Effect of ZBC260 treatment on tumorsphere formation and CSC marker expression in SUM149 cells co-cultured with macrophages. (A-C)** Tumorspehre co-culture with SUM149 tumor cells and THP-1 **(A)** M0-Like, **(B)** M1-Like, or **(C)** M2-Like **(D)** Gating scheme for flow cytometry analysis of ALDH^+^ population in macrophage and SUM149 co-culture. **(E)** Gating scheme for flow cytometry analysis of CD24^-^/CD44^+^ population in macrophage and SUM149 co-culture. **(F-K)** Effect of ZBC260 on the absolute number of ALDH^+^ and CD44^+^/CD24- cells in SUM149 cell line, n=3, **(F-H)** in 2D culture, and **(I-K)** 3D culture. Data points are the mean ±SEM for individual experiments. Data analysed by one-way ANOVA with Bonferroni's correction *P< 0.05; **P < 0.01; ***P < 0.001, ****P<0.0001.

**Supplementary Figure 9: Effect of ZBC260 on Stat6 and pStat6 in THP-1 macrophages.**

**(A-F)** Original, uncropped western blot membranes corresponding to the data presented in the main figures (Figure 4K-L**). (A–C)** Total STAT6 immunoblots from three independent biological replicates. Each panel represents a separate experiment using different biological samples. The identity of the samples loaded in each lane is indicated alongside the corresponding blot. **(D–F)** Phospho-STAT6 (p-STAT6) immunoblots from the same three independent biological replicates. Sample identities for each lane are indicated adjacent to the uncropped images. Molecular weight markers are shown (ladder) where applicable.
